# Distinct Inflammatory Macrophage Populations Sequentially Infiltrate Bone‐to‐Tendon Interface Tissue After Anterior Cruciate Ligament (ACL) Reconstruction Surgery in Mice

**DOI:** 10.1002/jbm4.10635

**Published:** 2022-05-31

**Authors:** Takayuki Fujii, Susumu Wada, Camila B. Carballo, Richard D. Bell, Wataru Morita, Yusuke Nakagawa, Yake Liu, Daoyun Chen, Tania Pannellini, Upneet K. Sokhi, Xiang‐hua Deng, Kyung Hyung Park‐Min, Scott A. Rodeo, Lionel B. Ivashkiv

**Affiliations:** ^1^ Arthritis and Tissue Degeneration Program and David Z. Rosensweig Genomics Research Center Hospital for Special Surgery New York NY USA; ^2^ Orthopaedic Soft Tissue Research Program Hospital for Special Surgery New York NY USA; ^3^ Department of Orthopaedic Surgery Tokyo Medical and Dental University Tokyo Japan; ^4^ Department of Medicine Weill Cornell Medicine New York NY USA; ^5^ BCMB allied program Weill Cornell Graduate School of Medical Sciences New York NY USA; ^6^ Graduate Program in Immunology and Microbial Pathogenesis Weill Cornell Graduate School of Medical Sciences New York NY USA

**Keywords:** SYSTEMS BIOLOGY ‐ BONE INTERACTORS, OSTEOIMMUNOLOGY, ANIMAL MODELS, ORTHOPAEDICS, INJURY/FRACTURE HEALING

## Abstract

Macrophages are important for repair of injured tissues, but their role in healing after surgical repair of musculoskeletal tissues is not well understood. We used single‐cell RNA sequencing (RNA‐seq), flow cytometry, and transcriptomics to characterize functional phenotypes of macrophages in a mouse anterior cruciate ligament reconstruction (ACLR) model that involves bone injury followed by a healing phase of bone and fibrovascular interface tissue formation that results in bone‐to‐tendon attachment. We identified a novel “surgery‐induced” highly inflammatory CD9+ IL1+ macrophage population that expresses neutrophil‐related genes, peaks 1 day after surgery, and slowly resolves while transitioning to a more homeostatic phenotype. In contrast, CX3CR1+ CCR2+ macrophages accumulated more slowly and unexpectedly expressed an interferon signature, which can suppress bone formation. Deletion of *Ccr2* resulted in an increased amount of bone in the surgical bone tunnel at the tendon interface, suggestive of improved healing. The “surgery‐induced macrophages” identify a new cell type in the early phase of inflammation related to bone injury, which in other tissues is dominated by blood‐derived neutrophils. The complex patterns of macrophage and inflammatory pathway activation after ACLR set the stage for developing therapeutic strategies to target specific cell populations and inflammatory pathways to improve surgical outcomes. © 2022 The Authors. *JBMR Plus* published by Wiley Periodicals LLC on behalf of American Society for Bone and Mineral Research.

## Graphical abstract

Sequential infiltration of repair tissue by distinct macrophage populations after ACL reconstruction surgery. Highly inflammatory macrophages expressing CD9 and sharing gene signatures with neutrophils infiltrate repair tissue rapidly and transiently after surgery. This is followed by influx of CX3CR1 and CCR2 positive macrophages that express an interferon signature. Targeting distinct macrophage populations may represent a therapeutic strategy to improve tissue repair and surgical outcomes.

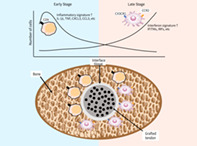

## Introduction

Tissue repair typically progresses through inflammatory, tissue formation and tissue remodeling phases.^(^ ^1^, ^2^ ^)^ The early inflammatory response typically lasts for 3 to 4 days and is characterized by influx of neutrophils and monocytes, and expression of canonical proinflammatory cytokines such as tumor necrosis factor (TNF) and interleukin 1 (IL‐1). The inflammatory phase sterilizes and “cleans up” the wound site and helps initiate the subsequent and partially overlapping phase of tissue formation, which occurs approximately 3 to 10 days postinjury. The tissue formation phase is characterized by resolution of classical inflammation and concomitant activation of mesenchymal cells, myofibroblasts, and angiogenesis. This results in the formation of fibrovascular granulation tissue and deposition of provisional extracellular matrix, which is subsequently remodeled by differentiating mesenchymal cells into a more organized and dense tissue. In many organs such as skin and lung, tissue formation and remodeling are promoted by the emergence of a type 2 immune response mediated by “M2‐like” macrophages and various T cell subsets^(^ ^3^, ^4^, ^5^ ^)^ that produce anti‐inflammatory, angiogenic, and growth factors, and activators of myofibroblasts and extracellular matrix (ECM) production such as transforming growth factor β (TGF‐β). The type 2 response in these tissues is associated with efferocytosis of apoptotic cells and promoted by the cytokines IL‐4 and IL‐13.^(^ ^6^, ^7^ ^)^ Less is known about factors and cell types that regulate healing of load‐bearing musculoskeletal tissues after injury or surgical repair.

The balance between the early inflammatory and subsequent type 2 immune reactions, and the kinetics of the transition from type 1 to type 2 inflammation, are important for effective wound healing and return to tissue integrity.^(^ ^1^, ^2^, ^5^ ^)^ Excessive or sustained type 1 inflammation, as occurs in type 2 diabetes, inflammatory diseases, or with lack of adequate wound site stabilization and resultant excessive mechanical forces on the tissue after surgical repair, results in delayed healing or chronic wounds.^(^ ^8^, ^9^, ^10^, ^11^ ^)^ Conversely, an excessive type 2 response, as occurs with elevated and prolonged IL‐4, IL‐13, or TGF‐β expression, can result in fibrosis and scarring that compromises tissue function.^(^ ^5^ ^)^ A major challenge is regeneration of specialized tissues, which is often mediated by stem cells, and in the musculoskeletal system is modulated by mechanical forces. The interaction of immune and stem cells is an emerging area of research,^(^ ^12^, ^13^ ^)^ but the immune cells and factors that regulate tissue regeneration, and the crosstalk between immune responses and mechanical forces, are not well understood.

Macrophages are innate immune cells that play important roles in host defense, tissue homeostasis, response to injury, and resolution of inflammation. In a longstanding model, macrophages “polarize” in response to environmental cues to assume various activation states.^(^ ^14^, ^15^ ^)^ Pro‐inflammatory cytokines such as TNF and interferon γ (IFN‐γ), often in combination with microbial or tissue damage products, induce a “classically activated” (“M1‐like”) state characterized by high expression of inflammatory factors such as TNF and IL‐1. In contrast, reparative and anti‐inflammatory factors such as IL‐4, IL‐13, and glucocorticoids induce various “alternatively activated” or “M2‐like” macrophages that produce suppressive and growth factors such as IL‐10, vascular endothelial growth factor (VEGF), and platelet‐derived growth factor (PDGF). After tissue injury, macrophages play a key role in the first two stages of tissue repair^(^ ^1^, ^2^, ^3^, ^4^, ^5^, ^16^, ^17^, ^18^, ^19^, ^20^, ^21^ ^)^; they produce inflammatory cytokines during the early phase, efferocytose apoptotic neutrophils during the transition, and subsequently develop a pro‐resolution phenotype that dampens inflammation and promotes angiogenesis, new tissue formation, and maturation. Recently, the macrophage polarization model has evolved as the importance of macrophage ontogeny (tissue or monocyte‐derived), of tissue‐specific phenotypes induced by local microenvironmental factors, and epigenetic programming has been increasingly appreciated.^(^ ^22^, ^23^, ^24^, ^25^, ^26^ ^)^ In addition, high dimensional analyses using flow/mass cytometry and single‐cell RNA sequencing (scRNAseq) of macrophages from various tissues under homeostatic, stress, or disease conditions have revealed novel macrophage subsets^(^ ^27^, ^28^, ^29^, ^30^ ^)^ with distinct functional phenotypes important for inflammation, repair, and fibrosis.

Anterior cruciate ligament reconstruction (ACLR) involves creating a mechanical bone injury by drilling a bone tunnel in articulating tibia and femur, followed by insertion of a tendon graft. An initial inflammatory phase with infiltration of neutrophils and monocytes/macrophages 1 to 7 days postoperatively is followed by an overlapping tissue repair phase that results in the formation of a highly cellular fibrovascular granulation tissue at the bone‐tendon interface at 7 days postoperation.^(^ ^31^, ^32^, ^33^ ^)^ A key aspect of healing is bone formation that occurs within or extends from underlying bone into the fibrovascular interface tissue.^(^ ^31^, ^34^, ^35^, ^36^, ^37^ ^)^ The interface tissue matures over the subsequent 3 weeks to become a more dense and organized fibrous tissue, with diminished cellularity and vascularity, and continuity of collagen fibers between bone and tendon. Close apposition of bone and tendon (ie, less intervening interface tissue) provides increased attachment strength and more secure healing. Repair failure, related in part to a diminished early healing response, remains a common problem and can lead to joint instability and posttraumatic osteoarthritis in up to 5% to 10% of cases.^(^ ^38^, ^39^ ^)^ Excessive or prolonged inflammation can adversely impact graft healing, attachment strength, and function, leading to poor healing with inferior strength at the graft‐to‐bone attachment site, and bone resorption around the tunnel (“tunnel widening”).^(^ ^40^, ^41^, ^42^, ^43^ ^)^ This inflammation can be mediated at least in part by macrophages that infiltrate interface tissue for at least 4 weeks post surgery^(^ ^32^, ^34^, ^35^, ^44^ ^)^; broad depletion of macrophages in a rat ACLR model using clodronate liposomes improved bone‐tendon healing by decreasing the volume of interface tissue, increasing new bone formation, and decreasing osteoclast‐mediated resorption around the bone tunnel.^(^ ^34^, ^35^, ^36^, ^44^ ^)^ However, a progressive increase in M2‐like macrophages beginning 7 days postoperatively^(^ ^32^ ^)^ suggested that macrophage subsets also likely have beneficial functions after ACLR, although little is known about macrophage phenotypes and factors that promote the healing/repair phase in this model.

In this study, we used a combination of scRNAseq, flow cytometry, and deep sequencing of flow‐sorted macrophages to characterize the immune/inflammatory response in bone‐to‐tendon interface tissue after ACLR. Our study revealed multiple macrophage subtypes that infiltrated interface tissues with distinct kinetics, whose phenotype was dynamically regulated during the transition from the early inflammatory to the tissue formation phase. Notably, a novel subset of CD9+ IL1+ CX3CR1− CCR2− highly inflammatory macrophages was rapidly induced after surgery, whereas CD9− CX3CR1+ CCR2+ macrophages that expressed IFN response genes accumulated with delayed kinetics. Deletion of *Ccr2* resulted in increased bone volume in the bone tunnel at the bone‐to‐tendon interface, implicating these cells, and possibly the IFN response, in suppressing bone formation and healing. Our study identifies novel macrophage subtypes that likely have distinct functions in post‐ACLR tissue repair, and suggests approaches to modulate the immune response to improve tissue healing and surgical outcomes.

## Materials and Methods

### Study design

We conducted an animal study using a murine ACLR model to identify the contribution of immune cell populations, especially macrophage subpopulations, to the tendon‐to bone integration by utilizing flow cytometry, scRNAseq, bulk RNA sequencing (bulk RNA‐seq), and histomorphology. Twelve‐week‐old C57/BL6 mice (*n* = 90 in total) underwent ACLR surgery. Cells infiltrated into the interface tissue were harvested at postoperative days (PODs) 1, 3, 7, and 14, and the cell populations and temporal dynamics in their transcriptome were assessed by scRNAseq. Complementary flow cytometry analysis was performed to define macrophage populations at PODs 1, 7, and 14. To analyze transcriptomic and morphologic differences among infiltrating macrophage populations at the bone‐tendon graft interface, cells from the interface tissue were harvested at PODs 1 and 14, sorted into subpopulations, and analyzed by bulk RNA‐seq and Giemsa staining. Bone remodeling and histological features of the interface tissues of wild‐type (WT) (*n* = 16) and CCR2‐deficient (*n* = 16) mice were assessed by micro‐computed tomography (μCT) and histology at POD 14 to determine the contributions of CCR2‐positive macrophages to tendon‐to bone integration.

#### Mice

All animal experiments were approved by the Weill Cornell Medical College IACUC. WT (C57BL/6J) and CCR2 KO mice (B6.129S4‐Ccr2tm1Ifc/J) were purchased from The Jackson Laboratory (Bar Harbor, ME, USA). Animals were housed in a specific pathogen‐free environment in the Weill Cornell Medicine vivarium.

#### ACLR

Twelve‐week‐old male mice underwent ACLR surgery as described.^(^ ^45^ ^)^ Briefly, mice were anesthetized and placed in a supine position. The skin of the medial side of the right ankle joint was incised longitudinally and the flexor digitorum longus tendon was identified and its proximal part was mobilized. The plantar area of the right hindpaw was incised longitudinally, and the flexor digitorum longus tendon was identified under the plantar fascia. The entire flexor digitorum longus tendon was delivered into the wound. A surgical clip (Synovis Micro Alliance, Birmingham, AL, USA; #GEM1521) was placed at the distal end of the tendon and the graft tendon was harvested by cutting at the distal end of the flexor digitorum longus. Next, the right knee joint was exposed using an anterior approach and the ACL was cut sharply using a scalpel. The bone tunnel was created by drilling using a 23G needle in the femur and the tibia, and then the graft was passed through the bone tunnel from the femur to the tibia. The proximal part of the graft was fixed extracortically by a surgical clip and the distal part was fixed by transosseous 4‐0 Ethibond suture (Ethicon, Somerville, NJ, USA) to the tibial shaft. The tendon was tensioned to 5 N during graft fixation. Postoperatively, animals received buprenorphine for pain management while comfort and recovery were assessed multiple times per day.

#### Cell isolation

Interface tissue surrounding the grafted tendon in the bone tunnel was harvested from the femoral and tibial tunnels using a 22G needle. The interface tissue was digested with Collagenase A (Sigma‐Aldrich, St. Louis, MO, USA), Dispase II (Sigma‐Aldrich), and DNAse I (Sigma‐Aldrich) for 15 minutes and then cells were filtered with a cell strainer. For isolating control bone and bone marrow cells, the distal femur and the proximal tibia were cut, minced, and enzymatically digested using the same protocol as used for the interface tissue. Erythrocytes were lysed with ACK lysis buffer (Sigma‐Aldrich).

#### Flow cytometric staining and sorting

Isolated cells were incubated with anti‐mouse CD16/CD32 antibodies for 10 minutes to prevent nonspecific binding of antibodies used for staining and then were stained with primary antibodies including anti‐mouse CD34, c‐Kit, Ter119, CD45, CD3, B220, Ly6C, Ly6G, CD14, CD115, F4/80, CCR2, CD64, CX3CR1, I‐A/I‐E, and CD11c (1:100), and CD11b (1:200) for 15 minutes in Brilliant Stain Buffer (BD Biosciences, San Jose, CA, USA; #563794), and for CD34, c‐Kit, and Ter119, samples were further stained with Streptavidin‐PerCP/Cy5.5 (1:500) for an additional 10 minutes. Stained cells were analyzed on a BD Symphony instrument (BD Biosciences) and data analysis was done using FlowJo software (Tree Star, Ashland, OR, USA). For cell sorting, CD11b+Ly6G+, CD11b+Ly6G−F4/80−, CD11b+Ly6G−F4/80+CD9+, and CD11b+Ly6G−F4/80+CX3CR1+ cells were sorted with Flow Cytometric Staining and Sorting (FACS) Aria II (BD Biosciences), following the gating strategy shown in Fig. 4*A*. In this sorting strategy the rare CD9+CX3CR1+ cells fell into the CD9+ sorted cell group. In pilot experiments fluorescence minus one (FMO) staining was performed to confirm proper gating and compensation. All antibodies were purchased from BioLegend (San Diego, CA, USA), and BD Biosciences. Antibody panels and information on antibodies are listed in Table S2.

#### Giemsa staining

At least 2000 sorted cells were loaded and immobilized on glass slides using Cytospin (Thermo Fisher Scientific, Waltham, MA, USA). Cells were stained with modified Giemsa staining solution (Sigma‐Aldrich; #GS500) and observed with a microscope.

#### Bulk RNA sequencing library preparation and data processing

Sorted cells were lysed and RNA was extracted with RNeasy Plus Micro Kit (Qiagen, Germantown, MD, USA). SMART‐Seq Stranded Kit (Takara Bio, Tokyo, Japan) was used for library preparation according to manufacturer's protocol. All samples passed quality control analysis on a Bioanalyzer 2100 (Agilent, Santa Clara, CA, USA). Libraries were sequenced on an Illumina NovaSeq 6000 (Illumina, San Diego, CA, USA) in the Weill Cornell Medical College Genomics Resources Core Facility to generate paired‐end reads. Read quality was assessed with FastQC v0.11.6 (https://anaconda.org/bioconda/fastqc) and adapters trimmed using Cutadapt v1.15 (https://anaconda.org/bioconda/cutadapt). Reads were then mapped to the mouse genome (mm10; https://hgdownload.cse.ucsc.edu/goldenpath/mm10/) and reads in exons were counted against Gencode (M23; https://www.gencodegenes.org/mouse/releaseM23.html) with STAR Aligner v2.5.3a (https://anaconda.org/bioconda/star). Differential gene expression analysis was performed in R v3.5.1 (R Foundation for Statistical Computing, Vienna, Austria; https://www.r-project.org/) using edgeR v3.20.9. Genes with low expression levels (<3 counts per million [cpm] in at least one group) were filtered from all downstream analyses. The Benjamini‐Hochberg false discovery rate procedure was used to calculate q‐value. Differentially expressed genes (DEGs) were defined as genes with q‐value <0.05 and log2 (fold‐change) ≥ 1. Principal component analysis (PCA) was performed using prcomp package in R. For hierarchical clustering dendrogram, cpms from 12089 genes were clustered by Euclidean method and linked with Ward.D method. DEGs in comparisons of CD9 day 1 versus CD9 day 14, CX3CR1 day 1 versus CX3CR1 day 14, CD9 day1 versus CX3CR1 day 1, and CD9 day 14 versus CX3CR1 day 14 were determined and 2558 genes were identified in total. Heat maps were generated from the averaged cpm using Pheatmap v1.0.12 (https://cran.r-project.org/web/packages/pheatmap/index.html).

#### scRNAseq library preparation and data processing

A cell suspension pooled from 10 to 15 mice was stained with monoclonal antibody Ter119 and 4′,6‐diamidino‐2‐phenylindole (DAPI). Ter119 and DAPI negative cells were sorted with Influx (BD Biosciences), and 25,000 cells were immediately loaded onto the Chromium Controller using Single Cell 3′ v3 Reagent following the manufacturer's protocol (10× Genomics, Pleasanton, CA, USA). All samples passed quality control analysis on a Bioanalyzer 2100 (Agilent). Paired‐end reads were obtained on an Illumina HiSeq 4000 in the Weill Cornell Epigenomics Core Facility or NovaSeq 6000 (Illumina) in the Weill Cornell Medical College Genomics Resources Core Facility. Reads were mapped and reads in exons were counted to the mouse genome (mm10) using CellRanger v3.0 (10× Genomics). Cells with gene number <300 and mitochondrial gene >95 percentile were filtered out from each dataset. After filtering, scaling normalization by deconvolving size factors were performed by Scran v1.12.1 (https://bioconductor.org/packages/release/bioc/html/scran.html) and doublets were excluded using scDblFinder v1.0 (https://bioconductor/.org/packages/release/bioc/html/scDblFinder.html). Gene count matrix of all datasets was integrated with Seurat v.3 (https://cran.r-project.org/web/packages/Seurat/index.html) to remove batch effects across different samples. As expected based on different efficiency of droplet‐mediated capture of hematopoietic and stromal cells on the 10× Genomics platform, >95% captured cells were hematopoietic cells.^(^ ^46^, ^47^ ^)^ The small number of captured stromal cells was excluded from the analysis. Cell cycle score was calculated using the CellCycleScoring function of Seurat and was regressed out during data scaling.^(^ ^48^ ^)^ In parameter settings, the first 40 dimensions of canonical correlation analysis (CCA) were used. Statistical inference of principal‐component analysis (PCA) was calculated by JackStraw function of Seurat, and the first 40 dimensions of principal‐components were used for dimensionality reduction by uniform manifold approximation and projection (UMAP). Clustering was performed using k‐Nearest Neighbor graph construction and Louvain community detection. SingleCellNet v0.1.0^(^ ^49^ ^)^ was used for unbiased computational cell‐type classification. Data from the Tabula Muris project was used as a training dataset.^(^ ^50^ ^)^ Pseudotime analysis was performed using Slingshot v 1.6.1 (https://bioconductor.org/packages/release/bioc/html/slingshot.html). The interferon‐stimulated gene (ISG) score was calculated based on the expression level of *Rsad2*, *Ifit2*, *Ifit3*, *Cmpk2*, *Cxcl10*, *Irf7*, *Isg15*, *Oasl1*, *Mx1*, and *Usp18* as described.^(^ ^51^ ^)^

#### μCT analysis

Bone ingrowth into the tendon‐bone interface tissue was evaluated by μCT in POD 28 samples. After euthanizing mice, the right knee joint was dissected and the soft tissue was removed. The right knee joint was fixed with 10% formalin for 3 hours. μCT imaging of the bone tunnel was assessed using a Scanco micro‐CT‐35 (Scanco Medical, Bruttisellen, Switzerland) with an isotropic voxel resolution of 6 μm (55 kVp, 145 μA, 400 ms integration time). The amount of newly formed bone within the tunnel since surgery was assessed by identifying a cylindrical volume of interest with a diameter of 0.64 mm (the same diameter as the 23G needle used to create the tunnels) and the total bone volume fraction (BV/TV) in the femoral and tibial bone tunnels was measured.

#### Histological analysis

Mice were euthanized using CO_2_ asphyxiation at POD 14. Specimens were fixed in 10% formalin solution followed by decalcification for one day (Immunocal; Decal Chemical Corp, Tallman, NY, USA). The knee joint was dissected, and the femoral and tibial component were separated and embedded in two different histological paraffin blocks. Each block was cross sectioned and stained in hematoxylin and eosin. Histology slides were reviewed by a pathologist at HSS with training in musculoskeletal pathology in a blinded manner. At least three serial cross‐sections from the tibia and the femur per mouse were evaluated. To assess the graft healing, the sections were scored for the following parameters: (i) graft remodeling, which describes the amount of tendon that was reconnected to the adjacent bone, directly or through fibrous tissue; (ii) interface fibrovascular component, describing the fibrovascular tissue bridging between the wall of the tunnel and the grafted tendon; (iii) angiogenesis and inflammation, mainly within the interface fibrovascular component; (iv) tendon degeneration, describing the presence of necrosis, separation and disorganization of collagen fibers, cystic degeneration; (v) ossification, intended as the formation of new bone around the tunnel. Remodeling, fibrovascular component, tendon degeneration, and ossification were scored from 0 to 3 based on the % of the tunnel circumference involved: 0% = score 0, 1–25% = score 1, 26–50% = score 2, and 51–100% = score 3. Angiogenesis and inflammation were arbitrarily scored as: absent = score 0, mild = score 1, moderate = score 2, and abundant = score 3.^(^ ^52^, ^53^ ^)^

#### Statistical analysis

All statistical analyses were performed with Prism 7.0 software (GraphPad Software, La Jolla, CA, USA) or R (ver. 3.5.0) using the two‐tailed, unpaired *t* test (two conditions), one‐way or two‐way ANOVA for multiple comparisons (more than two conditions) with post hoc Tukey's correction for multiple comparisons. Shapiro‐Wilk test was used to check normality. For all experiments: n.s, not significant, *p < 0.05, **p < 0.01, ***p < 0.005, and ****p < 0.001.

## Results

### scRNAseq identifies distinct CD9+ and CX3CR1+ CCR2+ macrophage populations in ACLR interface tissue

**Fig. 1:**
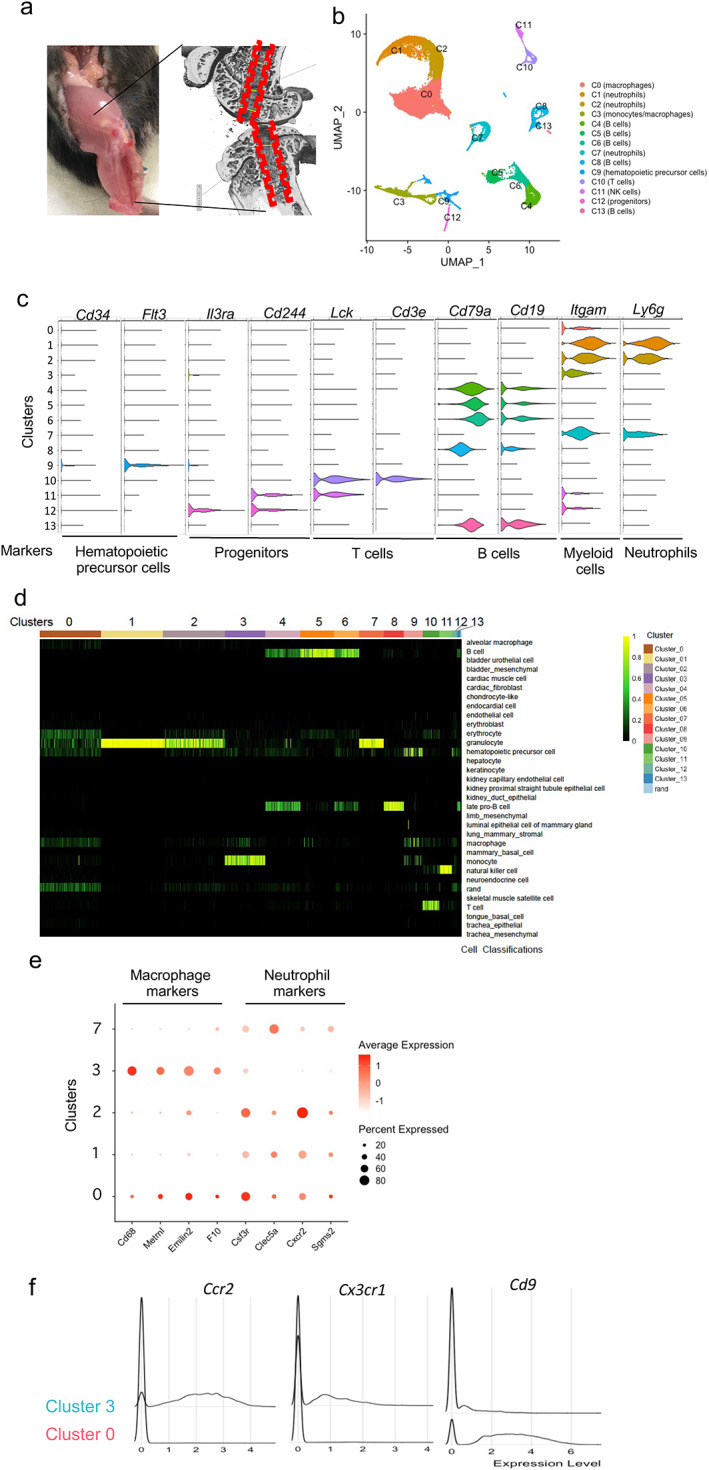
Single‐cell transcriptome profiling of the interface tissue after ACLR surgery. Cells were isolated from the interface tissue on day 1, 3, 7, and 14 after ACLR surgery and cells from 10 to 15 mice were pooled for each time point. (*A*) Schematic showing the area of harvesting of the interface tissue; data reflect integration of all time points. (*B*–*F*) Analysis of single cell RNAseq data using Seurat using cells isolated from the interface tissue on day 1, 3, 7, and14 after ACLR surgery. (*B*) UMAP projection of scRNAseq data. (*C*) Violin plots showing marker genes of different cell types. Markers used: Kit and Flt3, hemopoietic stem cells. Il3ra and Cd244, Progenitors. Lck and Cd3e, T cells. Cd79a and Cd19, B cells. Itgam, myeloid cells. Ly6g, neutrophils. (*D*) Unbiased cell identity classification by singleCellNet. (*E*) Expression of macrophage and neutrophil markers in clusters 0, 1, 2, 3 and 7. Macrophage markers: Cd68, Metrnl, Emilin2, and F10. Neutrophil markers: Csf3r, Clec5a, Cxcr2, and Sgms2. f, Single cell histogram of Ccr2, Cx3cr1, and Cd9 expression in clusters 0 and 3.

To better understand mechanisms by which macrophages regulate graft tendon‐to bone integration after ACLR surgery, we set out to define the changes in macrophage phenotypes over time as healing proceeds at the healing bone‐tendon graft interface using our murine ACLR model.^(^ ^45^ ^)^ The ACL was reconstructed in right knees by inserting the flexor digitorum longus tendon into femoral and tibial bone tunnels, simulating standard ACL reconstruction techniques used in patients.^(^ ^45^ ^)^ We then harvested bone‐tendon fibrovascular interface tissue and adjacent bone (Fig. 1*A*), as described in Materials and Methods, at PODs 1, 3, 7, and 14 after ACLR surgery and analyzed immune cell populations and how they evolve over time. In pilot experiments using flow cytometry to characterize interface tissue cells we found that approximately 50% of cells were of hematopoietic origin (CD45+) and of these 70% were of CD11b+ myeloid lineage comprised of neutrophils and macrophages (Fig. S1 *A*–*C*).

We then performed scRNAseq on interface tissue obtained 1, 3, 7, and 14 days after surgery. For these experiments we pooled cells from 10 to 15 mice/time point, flow sorted for live/non‐erythrocytic cells (DAPI− Ter119−) and obtained gene expression profiles for a total of 46,754 cells (15,339, 9574, 11,724, and 10,117 cells, on PODs 1, 3, 7 and 14, respectively) (Table S1). The tissue harvesting procedure (Fig. 1*A*) captures the fibrovascular tissue between tendon and bone and a sliver of underlying bone and thus reflects the cellular composition of newly formed soft interface tissue and underlying reactive bone that integrates with this soft tissue over time; as a control and comparison point we sequenced 8704 cells obtained from the same tibial and femoral bone regions in non‐operated mice. For technical reasons and cell number limitations the soft tissue and bone components of interface tissue could not be further separated. After accounting for batch effects among all datasets (Materials and Methods), nonlinear dimensional reduction by UMAP and graph‐based clustering of pooled cells from control and POD 1, 3, 7, and 14 samples identified 14 transcriptionally distinct clusters of interface tissue cells (Fig. 1*B* shows integration of all time points; Fig. S1 *D* shows cell clusters at individual time points). The 10× Genomics platform that we used effectively captures hematopoietic but not mesenchymal cells (Materials and Methods) and the small numbers of captured stromal cells were not further analyzed. Manual annotation based on key marker gene expression identified various hematopoietic cell types (Fig. 1*B*,*C*). This initial analysis identified five myeloid cell clusters, of which three (clusters 1, 2, and 7) were classified as neutrophils based on Ly6G expression, and two (clusters 0 and 3) were classified as macrophages based expression of *Itgam* (encoding CD11b) and other macrophage markers including *Spi1* (encoding master lineage‐determining factor PU.1), *Adgre1* (encoding F4/80), *Csfr1*, *Cd14*, and *Cd68* (Fig. 1*C*, Fig. S2). In contrast, neutrophils expressed *Ly6g*, master regulator *Cebpe* and other neutrophil marker genes such as *Mmp8* and *Mgam* (Fig. S2). Cells were similarly classified when using an unbiased approach of computational classification with SingleCellNet^(^ ^49^ ^)^ (Fig. 1*D*). SingleCellNet classified clusters 0 and 3 as macrophages, although cluster 0 also exhibited similarity to granulocytes (Fig. 1*D*) and expressed both macrophage and select neutrophil genes (Fig. 1*E*); cluster 0 was clearly distinguished from neutrophils based on morphology and additional bulk RNA‐seq (see Fig. 4 below). Cluster 3 also showed a strong similarity to monocytes (Fig. 1*D*). In accord with this classification system, cluster 3 expressed monocyte‐related genes *Cx3cr1* and *Ccr2*, whereas cluster 0 was negative for these markers but could be clearly distinguished from cluster 3 based on expression of *Cd9*, recently shown to be expressed in macrophages during inflammation and tissue repair^(^ ^30^, ^54^ ^)^ (Fig. 1*F*, Fig. S3 *A*). Thus, scRNAseq separated interface macrophages into two broad groups, CX3CR1+ CCR2+ and CD9+ macrophages.

**Fig. 2:**
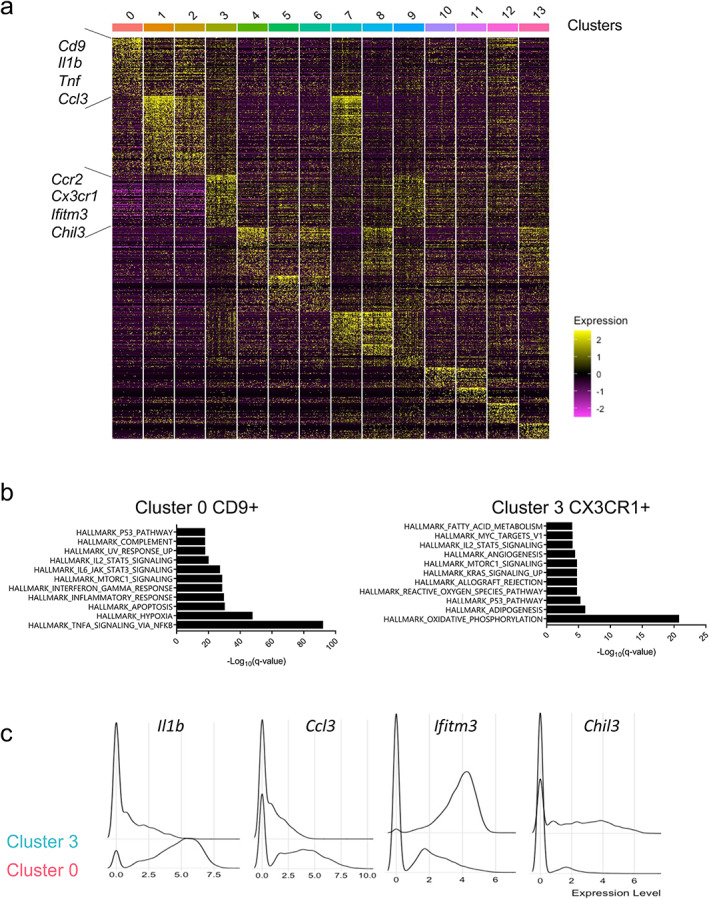
Subpopulations of macrophages that infiltrate interface tissue after ACLR surgery. (*A*) Heat map showing top genes enriched in each cluster obtained in Fig. 1. (*B*) GSEA of the marker genes of Cd9+ cells in cluster 0 and Cx3cr1+ Ccr2+ cells in cluster 3 as identified in *A*. (*C*) Single cell histogram of *Il1b*, *Ccl3*, *Ifitm3*, and *Chil3* in clusters 0 and 3. GSEA = gene set enrichment analysis.

To gain insight into functional differences between cluster 0 and cluster 3, we identified the 400 top differentially expressed marker genes in each cluster (Fig. 2*A*) and performed pathway analysis. Strikingly, CD9+ cluster 0 showed highly significant enrichment of canonical inflammatory nuclear factor κB (NF‐κB) and IFN‐γ pathways typical of “M1” classical inflammatory macrophages (Fig. 2*B*). The two macrophage clusters were also clearly separated by cellular metabolic pathways, with CD9+ macrophages showing a hypoxia response that is associated with glycolytic metabolism, mammalian target of rapamycin complex 1 (mTORC1), and inflammatory activation, whereas cluster 3 CX3CR1+ CCR2+ cells showed enrichment of oxidative phosphorylation pathways, which can be associated with either inflammatory “M1” or reparative macrophage functions (Fig. 2*B*).^(^ ^55^, ^56^, ^57^ ^)^ In line with this analysis, a large fraction of cluster 0 macrophages highly expressed inflammatory genes such as *Il1b* and *Ccl3* (Fig. 2*C*). In contrast, only a small subset of cluster 3 macrophages expressed *Il1b* and *Ccl3*, and expressed these genes at lower levels. Cluster 3 instead preferentially expressed select “M1” and “M2” genes such as, *Ifitm3* and *Chil3*, respectively (Fig. 2*C*, Fig. S3 *B*), but at this resolution of analysis a clear statistically significant pro‐inflammatory or anti‐inflammatory overall phenotype was not apparent (Fig. 2*B*). Overall, these results, based on analysis of cells pooled from multiple postsurgical time points, identify in ACLR interface tissue a highly inflammatory macrophage subset that shares gene expression pattern with neutrophils, and a distinct macrophage subset that shares gene expression pattern with monocytes and utilizes oxidative phosphorylation for cellular metabolism.

### Multiple macrophage subsets infiltrate interface tissue with distinct kinetics after ACLR

**Fig. 3:**
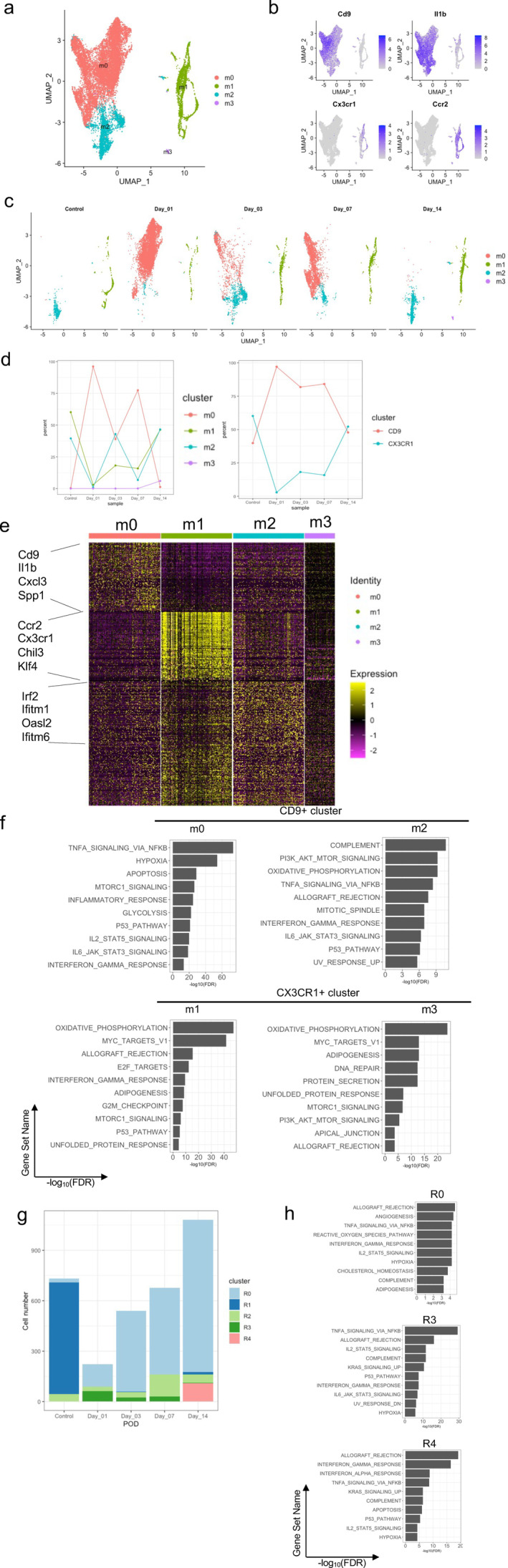
Identification of monocyte/macrophage subpopulations in the interface tissue after ACLR surgery. (*A*) UMAP projection of cluster 0 and 3 myeloid lineage cell populations on day 1, 3, 7, and 14 after ACLR surgery and from control tissue from non‐operated mice. (*B*) Expression pattern of *Cd9*, *Cx3cr1*, *Il1b*, and *Ccr2*. (*C*) UMAP showing clustering of macrophages in naïve bone (control) and in interface tissue at each time point (days 1, 3, 7 and 14 postsurgery). (*D*) Left panel: Changes in the percentages of m0–m4 clusters in the interface tissue. Right panel: Change in the percentages of cell types (m0 and m2 for CD9, and m1, m3 and m4 for CX3CR1). (*E*) Heat map of DEGs that serve as markers of clusters m0–m4. Top 200 genes are shown. (*F*) Pathway analysis of clusters m0–m3 using GSEA. (*G*) The number of cells in each subcluster of *Cx3cr1+ Ccr2+* cells (R0–R4, as defined in Fig. S4) on POD 0, 1, 3, 7, and 14. (*H*) GSEA pathway analysis of clusters R0, R3, and R4.

Recent research has highlighted macrophage heterogeneity, multiple phenotypic subtypes, and evolution of functional phenotype over time.^(^ ^58^ ^)^ Thus, we next focused our analysis on macrophages (clusters 0 and 3) to detect potential subsets and their kinetics of tissue infiltration; this analysis was performed using cell cycle regression because a fraction of CX3CR1+ CCR2+ cells expressed G2/M genes. Dimensionality reduction using UMAP and cell clustering of macrophages confirmed clear separation of *Cd9*+ *Il1b*+ and *Cx3cr1*+ *Ccr2*+ macrophages (Fig. 3*A*,*B*). Four macrophage clusters, termed m0–m3, were identified; this nomenclature does not correspond to the M1–M2 terminology for macrophage polarization. Clusters m0 and m2 expressed *Cd9* and *Il1b* and clustered together, whereas clusters m1 and m3 expressed *Cx3cr1* and *Ccr2* and clustered in a different region of UMAP space (Fig. 3*A*,*B*). Analysis of the presence of macrophage clusters over time (Fig. 3*C*) revealed that cluster m0 was minimally present in control bone/bone marrow tissue, massively increased on day 1 after ACLR surgery, and then gradually diminished during the healing process (Fig. 3*C*,*D*); m0 was the predominant *Cd9/Il1b*‐expressing cell cluster. The smaller *Cd9*‐expressing cluster m2 was present in control tissue, increased in interface tissue at POD 3, and was maintained until POD 14. *Cx3cr1*‐expressing clusters m1 and m3 were present in control bone/bone marrow tissue; these clusters exhibited a progressive but modest increase in interface tissue over time until POD 14, with m1 representing the predominant *Cx3cr1*‐expressing cluster (Fig. 3*C*,*D*). These results reveal that *Cd9*‐expressing macrophage subsets are rapidly induced during the early inflammatory response to ACLR surgery, whereas *Cx3cr1*‐expressing macrophages accumulate more slowly over time.

To gain insight into functional differences among the four macrophage clusters, we performed differential gene expression analysis. A total of 1799 genes showed significant differences in expression across the clusters. A heat map of expression the top 200 genes in each cluster is depicted in Fig. 3*E*; a gene list of cluster marker genes is provided in Appendix S1. Visual inspection of the *Cd9*‐expressing m0 cluster marker genes (Appendix S1) revealed numerous inflammatory genes including cytokines (eg, *Il1b*), chemokines (eg, *Cxcl3*, *Ccl3*, *Cxcl2*), Toll‐like receptors (*Tlr2*), and hypoxia‐inducible genes (eg, *Hilpda*, *Mif*). Accordingly, gene set enrichment analysis (GSEA) of m0 marker genes showed highly significant enrichment of inflammatory pathways such as TNF‐α signaling, inflammatory response, and interferon/interleukin‐Janus kinase‐signal transducer and activator of transcription (Jak‐STAT) signaling (Fig. 3*F*). Interestingly, the glycolysis pathway, which is a hallmark of inflammatory macrophage activation, was highly upregulated (Fig. 3*F*). *Cd9*‐expressing cluster m2 showed upregulation of distinct metabolic and complement pathways, similar inflammatory pathways (Fig. 3*F*), but in addition expressed a distinct module of marker genes that included interferon‐stimulated genes (ISGs) such as *Ifitm1*, *Ifitm6*, *Stat1*, and *Irf2* (Fig. 3*E*, Appendix S1).

The *Cx3cr1*‐expressing m1 and m3 clusters showed enrichment of oxidative phosphorylation genes, Myc pathway, and mTORC1 signaling, which is associated with anabolic metabolism, macromolecule biosynthesis and protein translation, and has been implicated in macrophage “training” and activation^(^ ^59^, ^60^ ^)^ (Fig. 3*F*). Although the inflammatory phenotype of these cell clusters was difficult to ascertain, possibly related to limited depth of sequencing and high expression of numerous metabolic genes (Appendix S1), the dominant m1 cluster showed highly significant enrichment of the “interferon gamma response” pathway (Fig. 3*F*; see also analysis of bulk RNAseq below). Thus, these results reveal macrophage subclusters that express distinct gene modules which can confer unique functional properties; eg, the canonical inflammatory response expressed by m0 and the IFN response expressed by m1 and m2. Overall, the data reveal a novel surgery‐induced inflammatory macrophage subset m0 that peaks during the early inflammatory phase, and the later emergence of macrophage clusters such as m1 and m2 that express an IFN signature.

**Fig. 4:**
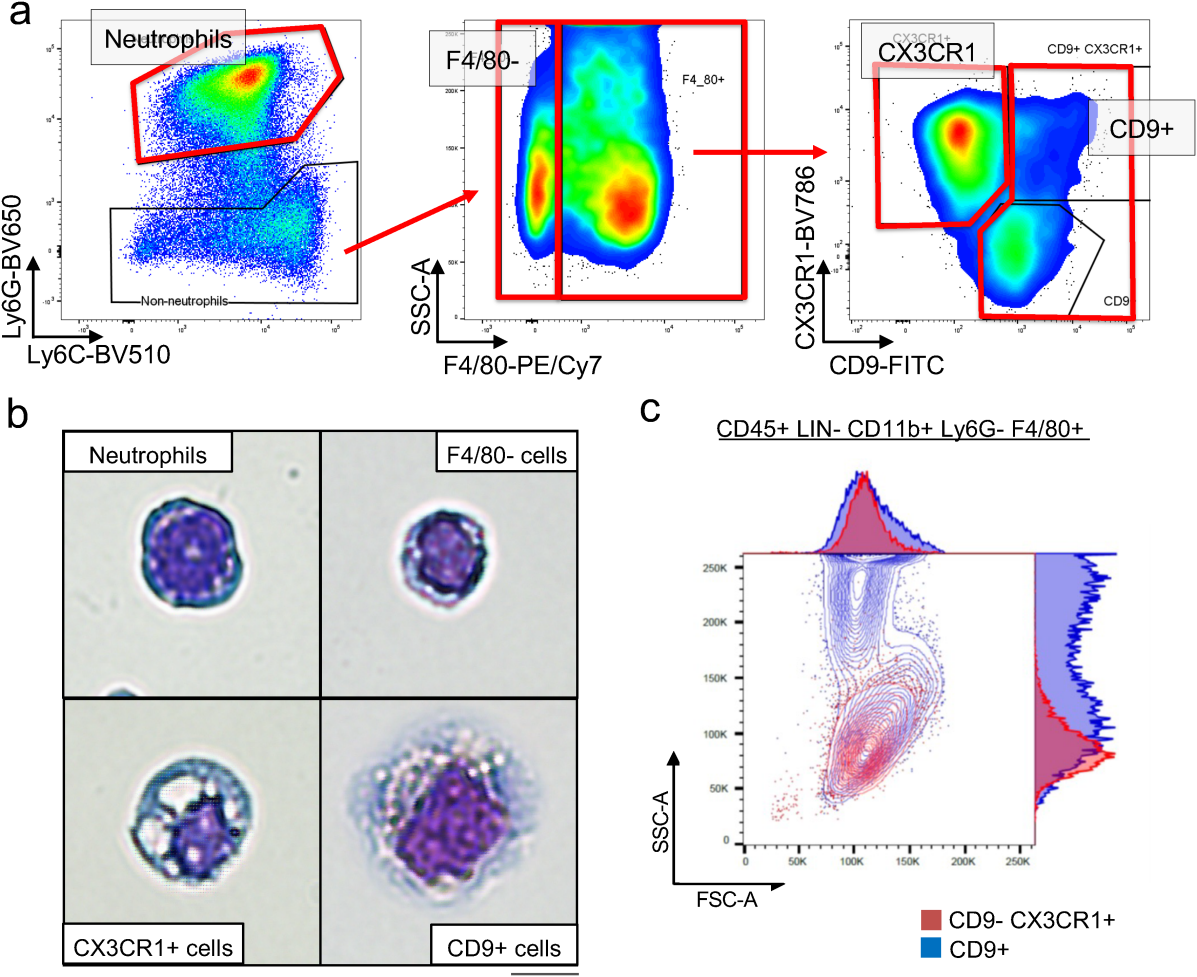
Flow cytometric and morphological analysis of distinct CD9+ and CX3CR1+ macrophages in interface tissue. Cells were isolated from the interface tissue on day 7 after ACLR surgery. (*A*) Representative flow cytometric plot on POD 7 showing the gating strategy for cell sorting. Boxes enclose sorted cells. (*B*) Photomicrographs of cells stained with May‐Grunwald Giemsa staining. Upper left quadrant = neutrophils; upper right quadrant = F4/80− cells; lower left quadrant = F4/80+ CD9− CX3CR1+ cells; lower right quadrant = F4/80+ CD9+ cells. Representative of at least 2000 cells analyzed. Scale bar: 10 μm. (*C*) A representative flow cytometric plot showing SSC (measures cell granularity) and FSC (measures cell size). FSC = forward scatter; SSC = side scatter.

To determine whether *Cx3cr1+ Ccr2*+ m1 and m3 cells contained inflammatory cell subsets, these cells were subclustered, which revealed five different subclusters, termed R0–R4, that evolved over time (Fig. S4 *A*,*B*, Fig. 3*G*). The dominant R1 subcluster in control bone did not exhibit inflammatory gene expression and disappeared rapidly after surgery. The dominant surgery‐induced R0 subcluster increased progressively over time and exhibited relatively weak enrichment of mixed inflammatory and IFN pathway genes (Fig. 3*H*). In contrast, surgery rapidly induced subcluster R3, which was strongly enriched in NF‐κB target genes and peaked on POD 1; subcluster R4 emerged on POD 14 and was strongly enriched in interferon response genes (Fig. 3*G*,*H*). Interestingly, cluster R3 expressed *Cd9* (Fig. S4 *C*) and may correspond to double positive cells detected by flow cytometry (see Fig. 4 below); cluster R2 was an unusual subcluster that corresponded to a minor macrophage population that expressed CX3CR1 but little CCR2 and also expressed CD9 and E2F target, G2M checkpoint, and MYC target genes, which are involved in cell proliferation and cell fate decision^(^ ^61^ ^)^ (Fig. S4 *D*). Ccr2 expression was maintained throughout the postoperative period (Fig. S4 *E*). Overall, the data show that *Cx3cr1+ Ccr2+* cells contain subsets of macrophages with distinct inflammatory profiles that are induced with different kinetics after surgery.

**Fig. 5:**
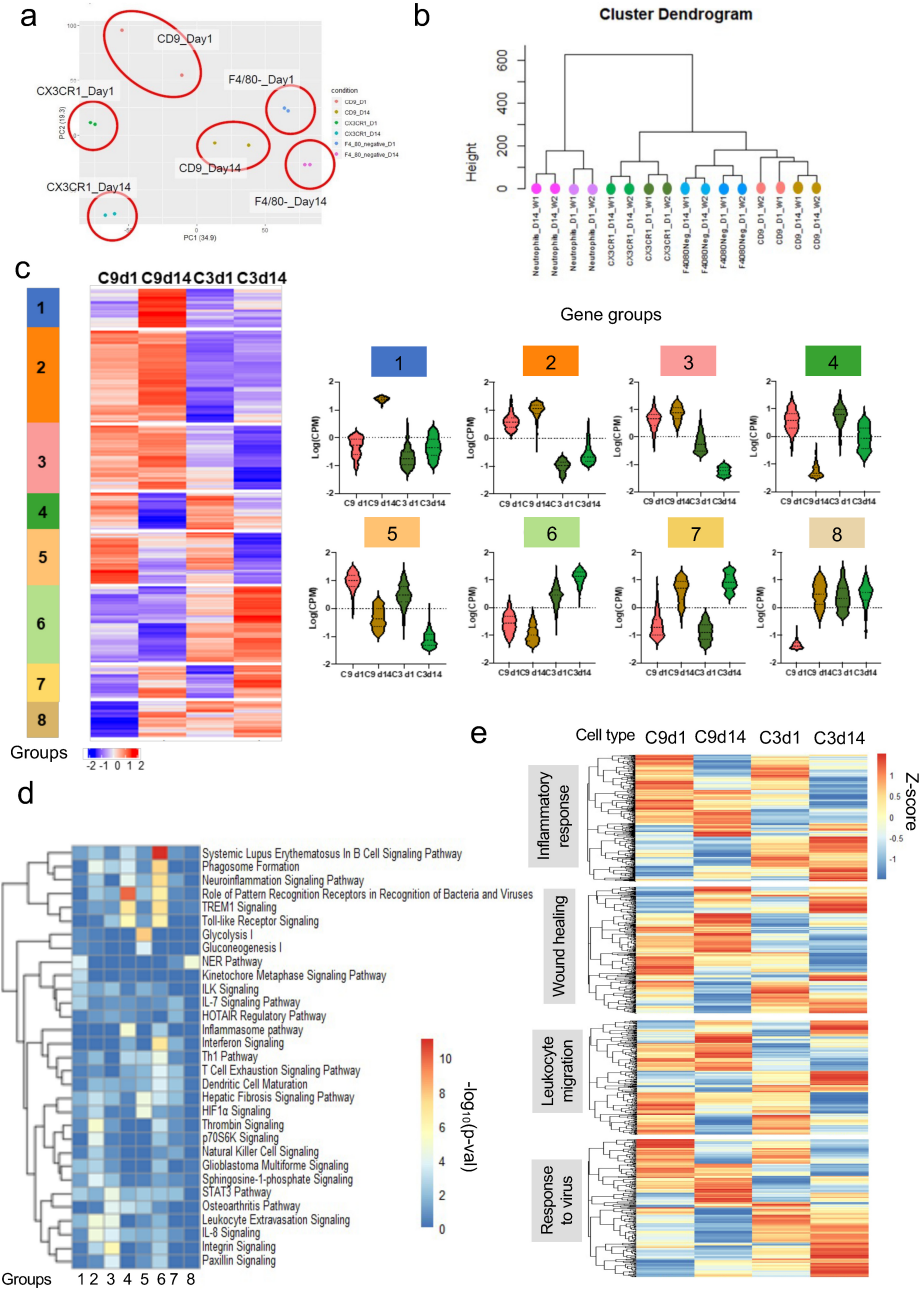
Bulk RNAseq reveals distinct transcriptomic features in subpopulations of myeloid lineage cells from the interface tissue. Cells from the interface tissue on day 1 and 14 after ACLR surgery were sorted (see Materials and Methods; *n* = 2, five to seven mice pooled for each sample). (*A*) PCA plot showing segregation of F4/80− cells, CD9+ macrophages, CX3CR1 macrophages on day 1 and 14 after ACLR surgery. (*B*) Cluster dendrogram. (*C*) Heat map showing expression of DEGs divided into 8 groups by k‐means‐clustering. Averaged CPM values are z‐score‐transformed. Right panels: violin plots of expression of genes in groups 1–8. (*D*) Pathway analysis using canonical pathway data sets of IPAs. (*E*) Heat map of expression of genes in inflammation, wound healing, leukocyte migration, and defense response to virus pathways. IPA = ingenuity pathway analysis.

An evolution of gene expression in transcriptionally defined clusters over time was also supported by a pseudotime analysis of *Cd9+ Il1b*+ macrophages (Fig. S5). We projected cells from clusters m0 and m2 onto PCA space and performed pseudotime analysis using Slingshot.^(^ ^62^ ^)^ In *Cd9*+ *Il1b*+ cells, pseudotime trajectories showed a clear trend along with physical‐time dynamics; *Cd9*+ cell phenotype evolved to day 7 after ACLR surgery with increased expression of inflammatory genes after surgery, and then returned toward a basal phenotype on POD 14 (Fig. S5 *A*–*C*). These results further support dynamic regulation of expression of subsets of genes within cell clusters during initiation and resolution of inflammation, which will be further investigated below in Fig. 5 using bulk RNAseq of sorted cell populations.

### CX3CR1+ and CD9+ macrophage populations identified by flow cytometry and morphology

One challenge in the single‐cell analysis field is the correspondence and alignment of cell clusters identified based on transcriptional profiling using scRNAseq with cell phenotypes defined based on conventional profiling of cell surface markers using flow cytometry.^(^ ^63^ ^)^ We next used flow cytometry to define macrophage populations in interface tissue; the gating strategy is shown in Fig. S6 *A*. Myeloid cells were defined as CD45+ Lin− (negative for T, B, NK, and red blood cell markers) CD11b+ cells that were further subdivided into Ly6G+ Ly6C+ neutrophils and Ly6G− Ly6C+ F4/80+ macrophages (Fig. 4*A*, Fig. S6 *A*). F4/80+ macrophages clearly separated into two predominant CX3CR1+ CD9− and CX3CR1− CD9+ populations on POD 7 (Fig. 4*A*, right panel), which were also observed on PODs 1 and 14 (Fig. S6 *B*). We also observed a minor CX3CR1+ CD9+ double‐positive macrophage population (Fig. 4*A*, Fig. S6 *B*); these cells likely correspond in part to the double positive clusters R2 and R3 described in Fig. S4 *B*,*C*, although review of the scRNAseq data also showed small numbers of *Cx3cr1*+ cells in the *Cd9*‐expressing cluster and vice versa (Fig. 3*B*). Similar flow cytometric results were obtained when staining for CCR2+ as for CX3CR1+ macrophages, although staining for cell surface CCR2 was often dim, presumably secondary to ligand‐mediated internalization^(^ ^64^ ^)^ (data not shown). CX3CR1+ and CD9+ cells were also observed in the CD45+ Lin−CD11b+ Ly6G−F4/80− cells (F4/80− myeloid cells) after ACLR surgery (Fig. S6 *C*). Thus, ACLR surgery induced macrophage populations in interface tissue that could be distinguished by cell surface CX3CR1 and CD9 expression.

The relatively clean separation of interface macrophages into CD9+ and CX3CR1+ populations enabled a flow‐sorting strategy (see Materials and Methods) to obtain purified cell populations, which we first applied to visualize cell morphology using Giemsa staining. We sorted CD11b+ myeloid cells from POD 1 and 14 interface tissue into Ly6G+ neutrophils, F4/80− myeloid cells (Fig. 4*A*, middle panel), F4/80+ CD9+ cells and F4/80+ CX3CR1+ cells (Fig. 4*A*, right panel). Each population exhibited distinct morphology (Fig. 4*B*), with Ly6G+ cells exhibiting a neutrophil morphology with a band‐like nucleus. F4/80− myeloid cells had a high nucleus‐to‐cytoplasm (N/C) ratio with limited nuclear polymorphism, suggestive of pro‐monocytes or immature monocytes.^(^ ^65^ ^)^ CX3CR1+ F4/80+ cells showed a macrophage‐like morphology with extensive cytoplasm, peripherally shifted nucleus, and cytoplasmic vesicles.^(^ ^66^, ^67^ ^)^ CD9+ F4/80+ macrophages had the largest cytoplasm and nucleus with multiple small vacuoles; accordingly, CD9+ macrophages exhibited large granularity by side scatter (Fig. 4*C*). These results further separate CD9+ macrophages from CX3CR1+ macrophages and neutrophils. Subpopulations of CD9+ and CX3CR1+ macrophages could be detected using a multiparameter flow cytometry approach (Fig. S7), although the relationship of these subpopulations to transcriptionally‐defined clusters defined by scRNAseq remains to be identified in future work.

### Transcriptomic analysis of purified CD9+ and CX3CR1+ macrophages

The scRNAseq analysis presented in Figs. 1, 2, 3 is limited by depth of sequencing and thus does not fully capture gene expression in each cell and cluster. We reasoned that the ability to purify macrophage subsets based on CD9 and CX3CR1 expression coupled with deep sequencing would enable a more comprehensive analysis of cell phenotype, in particular into potential functions of CX3CR1+ macrophages. Additionally, analysis of cells purified at different time points would provide a deeper analysis of evolution of gene expression over time, which was already apparent in the scRNAseq and pseudotime analysis. Thus, we performed bulk RNAseq on purified myeloid cell populations flow‐sorted from interface tissue as described in Fig. 4*A* and Fig. S6 *A*: Ly6G+ neutrophils, Ly6G−F4/80− myeloid cells, Ly6G−F4/80+ CD9+ macrophages, and Ly6G−F4/80+ CX3CR1+ macrophages. Samples were obtained on PODs 1 and 14 and cells from seven mice were pooled for each sort to obtain a data set of 16 samples, including biological replicates, derived from 30 mice. PCA, which depicts the overall variance between datasets, clearly separated neutrophils from macrophages and F4/80− myeloid cells along PC1 (which explained 48% of variance), and also revealed separation of day 1 and day 14 samples along PC2 (which explained 17% of variance) (Fig. S8 *A*). Thus, neutrophils and macrophages separated mostly based on cell type, although time after surgery also played a role.

PCA analysis of macrophage gene sets (Fig. 5*A*) clearly separated CD9+ macrophages, CX3CR1+ macrophages, and F4/80− myeloid cells along PC1; each cell type showed a similar separation between POD 1 and 14 samples along PC2. This separation of cell types was corroborated by hierarchical clustering, in which neutrophils occupied their own branch, and CD9+ and CX3CR1+ macrophages clearly segregated into distinct branches (Fig. 5*B*). Thus, this data set provides the capability for more extensive transcriptional profiling to identify functional differences between cell types and time points.

DEGs among CD9+ and CX3CR1+ macrophages on PODs 1 and 14 were clustered according to pattern of expression and visualized on a heat map (Fig. 5*C*). Several interesting patterns of expression (gene groups) of potential biological importance were apparent:

1. Genes that were elevated on POD 1 in both CD9+ and CX3CR1+ cells (gene groups 4 and 5) and decayed over time, thus representing a “day 1 signature” (Fig. 5*C*). Bioinformatic analysis revealed that the “day 1 signature” was highly enriched in genes in inflammatory Toll‐like receptor/TNF/inflammasome/IL‐1‐NF‐kB pathways (Fig. 5*D*, Fig. S8 *B*–*D*), which is in accord with an inflammatory environment shortly after surgery.^(^ ^32^, ^34^, ^35^, ^36^, ^44^ ^)^ Gene group 5, which was more highly expressed in CD9+ cells, was enriched for glycolysis and hypoxia pathway genes (Fig. 5*D*, Fig. S8 *C*,*E*), similar to the single cell analysis in Fig. 3*F*.
2. Genes preferentially expressed in CD9+ macrophages whose expression increased from day 1 to day 14 (groups 1 and 2). These groups were enriched in genes in growth factor and wound healing pathways (Fig. 5*D*,*E*, Fig. S8 *D*), suggesting that CD9+ macrophages can acquire a homeostatic/tissue repair component/gene module over time.
3. Genes that distinguish CD9+ from CX3CR1+ macrophages (group 3); this group was composed of inflammatory, chemotaxis‐related, and immune modulatory genes that maintain expression until day 14 (Fig. 5*D*,*E*, Fig. S8 *D*). Thus, the inflammatory nature of CD9+ cells had not entirely resolved by 14 days.
4. Gene group 6, which was preferentially expressed in CX3CR1+ cells and increased in expression from day 1 to day 14, exhibited a strong IFN signature most suggestive of type I IFN signaling in addition to inflammatory gene expression (Fig. 5*D*,*E*, Fig. S8 *B*–*E*); this result is in accord with the IFN signature observed in m1 cells in Fig. 3*F* and the R4 subset that emerged on POD 14 (Fig. 3*H*). Interestingly, the score for ISG expression increased in CX3CR1+ cells from day 1 to day 14 (Fig. S8 *F*). A strong IFN signature in POD14 CX3CR1+ macrophages was confirmed by a pairwise comparison to POD 14 CD9+ cells (Fig. S9 *A*,*B*), and the expression pattern of the full Hallmark‐defined ISG gene set is depicted in a heat map in Fig. S9 *C*. Overall, the bulk RNAseq results further delineate the functional separation of CD9+ and CX3CR1+ macrophages, show the attenuation of a canonical inflammatory response in both cell types over time, whereas an IFN response increases in CX3CR1+ macrophages over time.

### CCR2 deficiency increases bone mass in interface tissue

**Fig. 6:**
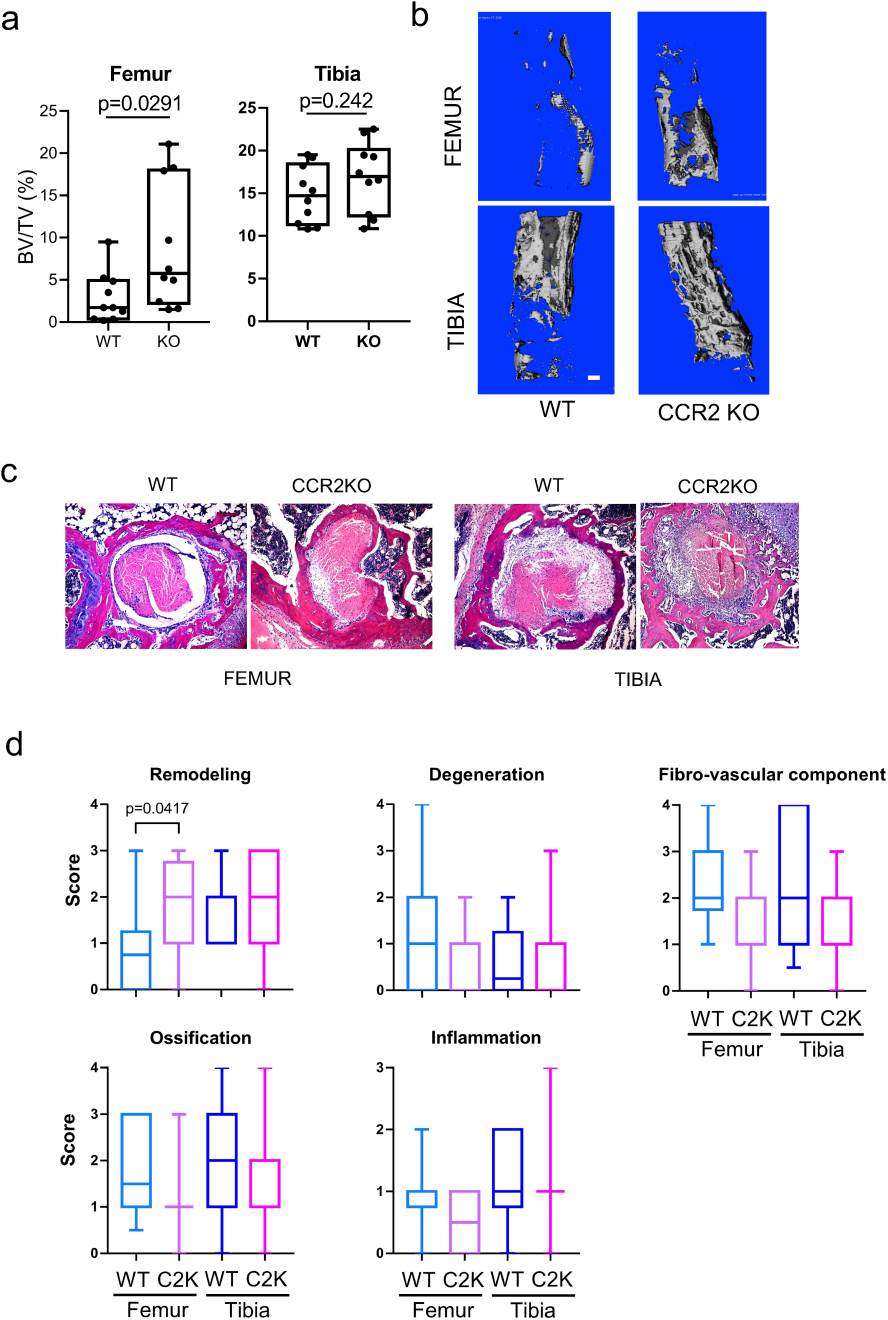
CCR2‐deficiency results in increased bone mass in the bone tunnel. The knee joints (WT = 10, KO = 9) were harvested on day 28 (*A*) and day 14 (*B*,*C*) after ACLR surgery. (*A*,*B*) μCT analysis. Left panel shows bone volume per tissue volume of newly formed bone in the bone tunnel. Right panel shows representative 3D images of the bone tunnel in the distal femur and the proximal tibia. Scale bars: 1 mm. (*C*,*D*) Histological analysis and scoring. Representative images are shown in *C*. Histology score is shown in *D* (WT: *n* ≥ 11, CCR2KO: *n* ≥ 15). *p < 0.05 by two‐tailed unpaired *t* test (*A*) or one‐way ANOVA with Tukey's post hoc test (*D*). KO = knockout.

Both CD9+ IL1+ and CX3CR1+ CCR2+ macrophages contributed to the early inflammatory phase after ACLR, but CX3CR1+ CCR2+ cells accumulated over time and expressed an increasing IFN signature. To begin to test the functional importance of CX3CR1+ CCR2+ cells, we used mice deficient in CCR2, which prevents recruitment of monocytes into the circulation and allows testing of the monocyte contribution to tissue repair. CCR2 deficiency will abrogate migration of many of the above‐described CX3CR1+ CCR2+ cells to sites of inflammation and tissue injury,^(^ ^68^ ^)^ although the small number of CX3CR1+ CCR2− cells will not be affected. Strikingly, CCR2 deficiency resulted in increased mass of new bone in the bone tunnels of the femur and to a lesser extent the tibia as assessed by μCT (Fig. 6*A*,*B*). In line with the increased bone mass, histological analysis showed that bone remodeling in the bone tunnel was significantly higher in CCR2‐deficient mice relative to WT mice (Fig. 6*C*,*D*). However, other histological parameters were comparable between WT mice and CCR2‐deficient mice (Fig. 6*D*). Thus, our data suggest that CX3CR1+ CCR2+ cells suppress accumulation of new bone around the tendon in the bone tunnel.

## Discussion

Immune cells play an important role in repair of various injured tissues such as skin and lung,^(^ ^1^, ^2^, ^3^, ^4^, ^5^ ^)^ but their role in healing of mechanically‐loaded musculoskeletal tissues is not well understood. We investigated functional phenotypes of macrophages in a mouse ACLR model that is characterized by bone injury followed by a healing phase of fibrovascular tissue and bone formation that results in bone‐to‐tendon attachment. A key finding of our study is identification of a highly inflammatory CD9+ IL‐1+ macrophage population that exhibits similarities in gene expression with neutrophils and is rapidly mobilized at the site of a bone injury that occurs adjacent to the bone marrow. ACLR induced infiltration of interface tissue by two broadly distinct populations of CD9+ IL‐1+ and CX3CR1+ CCR2+ macrophages with different kinetics and gene expression profiles. Highly inflammatory CD9+ IL1+ cells were the dominant macrophage population that peaked early after ACLR and diminished over time, concomitant with decreased expression of inflammatory genes and increased expression of growth factor and wound healing genes. In contrast, CX3CR1+ CCR2+ macrophages accumulated more slowly over time and unexpectedly expressed an IFN signature. scRNAseq revealed subsets within both CD9+ and CX3CR1+ macrophage populations, in accord with a complex evolving inflammatory response. CCR2+ macrophages were implicated in suppression of bone mass at the bone‐to‐tendon interface. These results reveal complex patterns of macrophage activation and inflammatory gene expression that can potentially be therapeutically targeted to improve healing and outcomes after ACLR.

In other injured tissues such as skin and lung that are distant from the bone marrow, both tissue‐resident and monocyte‐derived macrophages contribute to tissue repair.^(^ ^1^, ^2^, ^3^, ^4^, ^5^ ^)^ Monocytes, which are CX3CR1+ CCR2+, enter inflamed sites from the circulation and subsequently differentiate into macrophages. In our system, where injury is adjacent to bone and bone marrow (BM) and results in new tissue formation, there are no “tissue resident” macrophages per se in the newly formed interface tissue bridging underlying bone and the tendon graft. The de novo interface tissue in the bone tunnel can potentially be infiltrated by BM macrophages from underlying bone^(^ ^69^ ^)^ that can respond to injury‐induced chemokines and migrate directly to the adjacent site of injury, and by BM monocytes that can potentially migrate to the injury site either directly or via the circulation. Bone injury and tendon insertion elicited a dramatic and very early (peaking at 1 day) accumulation of large, granular macrophages that were highly inflammatory, exhibited glycolytic metabolism, and a gene expression profile with similarities to, but also clear differences from, neutrophils. To our knowledge, these “surgery‐induced macrophages” identify a new cell type in the early phase of inflammation, which in other tissues is dominated by blood‐derived neutrophils.^(^ ^1^, ^2^, ^3^, ^4^, ^5^ ^)^ The rapid appearance of these CX3CR1− CCR2− cells suggests direct migration and activation of BM macrophages, rather than differentiation from myeloid precursors or monocytes. The CD9+ macrophages contribute to the early inflammatory phase, but over time the phenotype of CD9+ macrophages evolves, with a decrease in inflammatory and an increase in growth factor and wound healing–related gene expression. Although it is not clear whether this change in phenotype is related to changes in gene expression by infiltrating cells, or to later phase migration and differentiation of newly infiltrating macrophages, it suggests that CD9+ macrophages have a plastic phenotype determined in part by the microenvironment, and can also participate in tissue repair.

In contrast to CD9+ macrophages that exhibit a de novo surgery‐induced phenotype, CX3CR1+ CCR2+ macrophages were observed in control tissues and were clearly distinguished from CD9+ macrophages by oxidative phosphorylation rather than glycolytic metabolism. Interestingly, subsets of CX3CR1+ CCR2+ cells, which likely represent cells within or adjacent to interface tissue, express an ACLR‐induced early inflammatory program, followed by a strong IFN response. In a sterile inflammatory setting after tissue injury, type I IFNs are most typically induced by activation of macrophage sensors of extracellular matrix degradation products generated during tissue remodeling (such as TLR4) or sensors of nucleic acids released by necrotic cells (such as TLRs 7–9).^(^ ^70^ ^)^ The role of IFN responses in tissue repair is not well understood, and IFNs have been reported to promote injury‐associated inflammation^(^ ^71^ ^)^ but also suppress collagen synthesis, fibroblast proliferation, and bone formation and remodeling.^(^ ^72^, ^73^ ^)^ CCR2 has been implicated in bone remodeling in physiological and pathological conditions. CCR2‐deficient mice exhibited increased bone mass with decreased numbers of osteoclasts.^(^ ^74^ ^)^ Although the role of CCR2 in osteoporosis‐induced bone loss is controversial,^(^ ^74^, ^75^ ^)^ the MCP‐1/CCR2 axis has also been shown to provide crucial signaling for recruitment of mesenchymal progenitor cells to fracture sites and CCR2 deficiency delayed fracture healing.^(^ ^76^ ^)^ Our findings suggest that bone mass inside the bone tunnel was increased in CCR2‐deficient mice compared to control mice. Further study will be needed to understand how CCR2 signaling contributes to the bone‐to tendon repair, including effects on bone mass of bone that surrounds the bone tunnel. Overall, it is tempting to speculate that the suppressive function of CX3CR1+ CCR2+ macrophages on bone formation at the bone‐to‐tendon junction is related to the IFN response, which may also have additional effects on tissue formation and remodeling in this system.

The healing interface tissue after ACLR did not exhibit canonical M2 macrophages that express IL‐4/IL‐13‐STAT6 target genes. IL‐4/IL‐13‐STAT6 signaling promotes repair by attenuating expression of inflammatory genes like *Il1b*,^(^ ^77^ ^)^ restraining IFN responses,^(^ ^78^ ^)^ promoting ECM remodeling,^(^ ^11^ ^)^ and driving differentiation of “M2‐like” macrophages that express trophic factors.^(^ ^5^ ^)^ The absence of a clear IL‐4/IL‐13 response in interface tissue is most likely explained by lack of cells that either produce or induce expression of IL‐4/IL‐13, such as innate lymphocytes, mast cells, eosinophils or basophils. These cell types are present and promote healing in epithelial and barrier tissues, and their low numbers or absence in many musculoskeletal tissues presents a challenge for these tissues to heal in the absence of IL‐4/IL‐13‐STAT6 signaling. Absence of an IL‐4 response may be permissive for development of a deleterious extended IFN response, as was observed after ACLR, and may result in a tissue formation process that is predominantly driven by growth factors and TGF‐β that lack some of the immunomodulatory and homeostatic features of an IL‐4–driven type 2 response.

Our study opens up new lines of research and therapeutic strategies for improving outcomes after ACLR. Because increasing bone formation is believed to improve fixation and prevent tunnel widening,^(^ ^34^, ^36^, ^79^, ^80^ ^)^ targeting pathways expressed by CX3CR1+ CCR2+ macrophages, such as the IFN response, represents a promising approach. It will be interesting to inhibit the IFN response at various time points after surgery using US Food and Drug Administration (FDA)‐approved Jak inhibitors^(^ ^81^ ^)^; this strategy may be more effective than CCR2 inhibition, because Jak inhibitors would also suppress the IFN response that was present in a subset of CD9+ macrophages. Given that the IFN response begins several days after surgery, initiation of therapy postoperatively could achieve the goal of increasing bone without suppressing the initial inflammatory response required for microbial clearance and initiation of the wound healing response. Broadly targeting CD9+ IL1+ macrophages throughout the time course of healing risks attenuating their potential pro‐healing functions at later time points, or overly compromising the initial inflammatory response. Alternative strategies would involve targeting some of their main inflammatory mediators, such as IL‐1, whose blockade has already been suggested to be efficacious in patients after ACLR.^(^ ^82^ ^)^

Our study has several limitations. A technical limitation is related to challenges in harvesting small interface tissues, which include a sliver of underlying bone and thus some of the cell types we identified could be present in bone surrounding the bone tunnel; the distribution of the macrophage subtypes in tissues and their potential interactions with stromal cells remain to be defined. The analysis of bone phenotype in CCR2‐deficient mice is limited and needs to be expanded in future work, including analysis of bone surrounding the bone tunnel. We focused on single cell analysis of isolated macrophages; it will also be important to compare our findings in a small animal model to macrophage phenotypes after ACLR in human subjects. An approach of sampling tissues shortly after ACLR in human subjects is precluded by ethical concerns related to potentially compromising the graft, but this issue could potentially be indirectly addressed by analyzing samples from patients who require revision surgery for graft failure.

In summary, we have characterized the inflammatory response after ACLR using high‐dimensional genomewide technologies. We have identified a novel “surgery‐induced” inflammatory CD9+ IL1+ macrophage population, and an IFN signature that increases over time and may suppress de novo bone formation and effective healing. This work sets the stage for developing approaches to therapeutically modulate immune responses to improve ACLR outcomes.

## AUTHOR CONTRIBUTIONS

**Takayuki Fujii:** Data curation; formal analysis; investigation; methodology. **Susumu Wada:** Data curation. **Camila B. Carballo:** Data curation; formal analysis; investigation; methodology. **Richard Bell:** Formal analysis; visualization. **Wataru D. Morita:** Data curation; formal analysis. **Yusuke Nakagawa:** Formal analysis. **Yake Liu:** Data curation. **Daoyun Chen:** Data curation. **Tania Pannellini:** Formal analysis; visualization. **Upneet K. Sokhi:** Formal analysis. **Xiang‐Hua Deng:** Methodology. **Kyung Hyung Park‐Min:** Formal analysis; methodology; visualization. **Scott A. Rodeo:** Methodology; supervision. **Lionel B. Ivashkiv:** Conceptualization; funding acquisition; project administration; supervision.

## Conflicts of interest

The authors declare that there are no conflicts of interest regarding the publication of this paper.

### PEER REVIEW

The peer review history for this article is available at https://publons.com/publon/10.1002/jbm4.10635.

## Supporting information


**Figs. S1–S9.** Supporting information

**Appendix S1.** Supporting information

**Tables S1–S2.** Supporting information

## Data Availability

The scRNAseq and bulk RNAseq datasets that were generated by the authors as part of this study have been deposited in the Gene Expression Omnibus database with the accession code GSE171480.
