# Supplementary material for: Distinct Inflammatory Macrophage Populations Sequentially Infiltrate Bone‐to‐Tendon Interface Tissue After Anterior Cruciate Ligament (ACL) Reconstruction Surgery in Mice
Source: JBMR Plus. 2022 May 31;6(7):e10635. doi: 10.1002/jbm4.10635 (PMC9289991; doi:10.1002/jbm4.10635)
Supplement: Supplementary file 1 — Figs. S1–S9. Supporting information [file JBM4-6-e10635-s003.pdf]

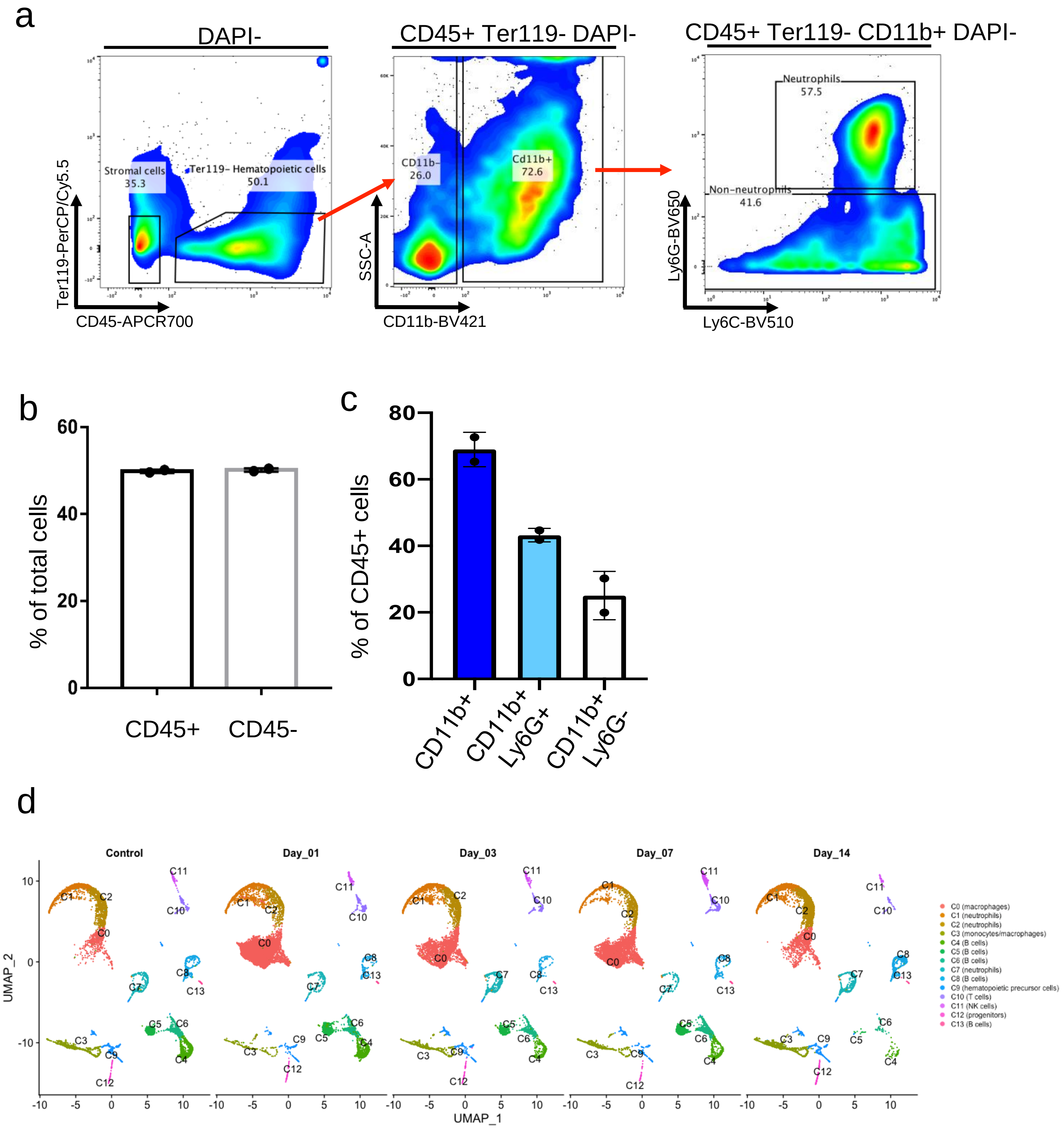

Supplementary Fig. 1 Immunophenotyping of immune cells in the interface tissue at POD 7.

a) Gating strategy for identifying CD45+ immune cells (CD45+,left panel), myeloid cells (CD11b+, middle panel), neutrophils (Ly6G+, Right panel) or non-neutrophils (Ly6G-). b, c) Percentage of CD45+ cells (b), CD11b+ myeloid cells, CD11b+ Ly6G+neutrophils and CD11b+Ly6G- non-neutrophils (c). d) UMAP projection of scRNAseq data corresponding to Fig. 1b but showing each time point individually.

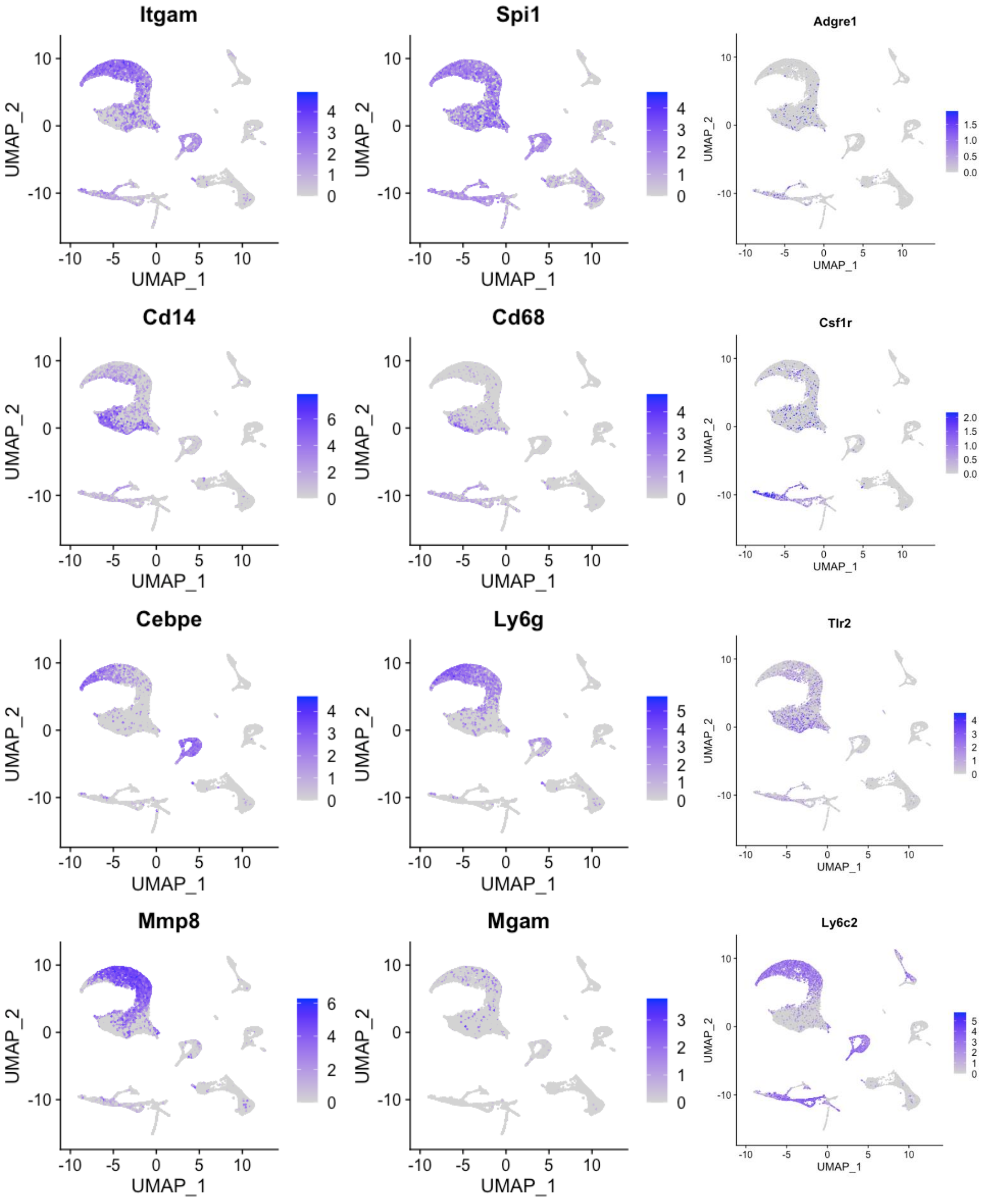

Supplementary Fig. 2 UMAP plot showing single cell transcriptional profiling of representative marker genes.
Color scale: natural log-scaled normalized counts.

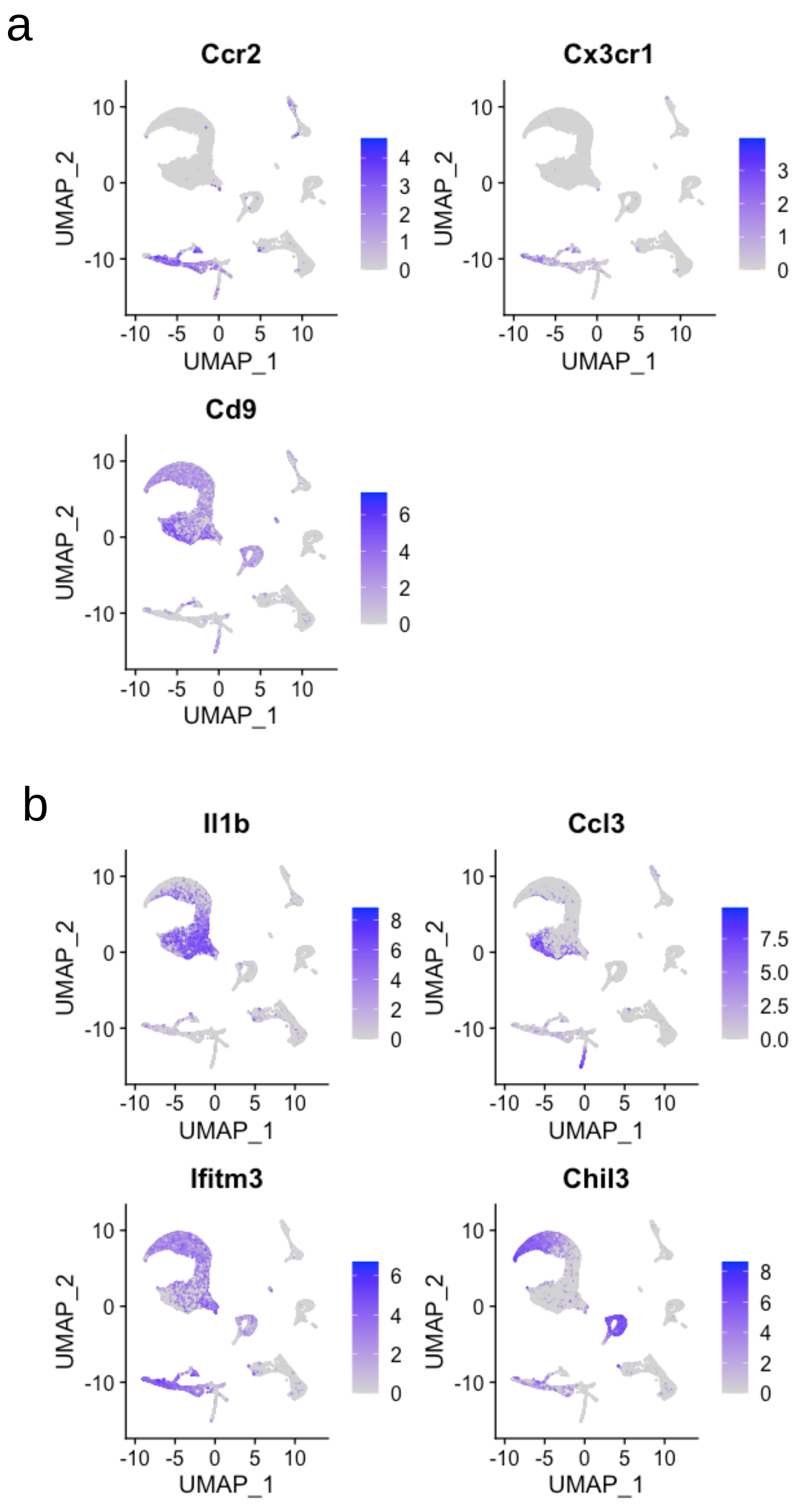

Supplementary Fig. 3. Feature plots showing gene expressions
a) Feature plots of *Ccr2, Cx3cr1* and *Cd9*. b) Feature plots of *Il1b* ,*Ccl3, Ifitm3* and *Chil3*.

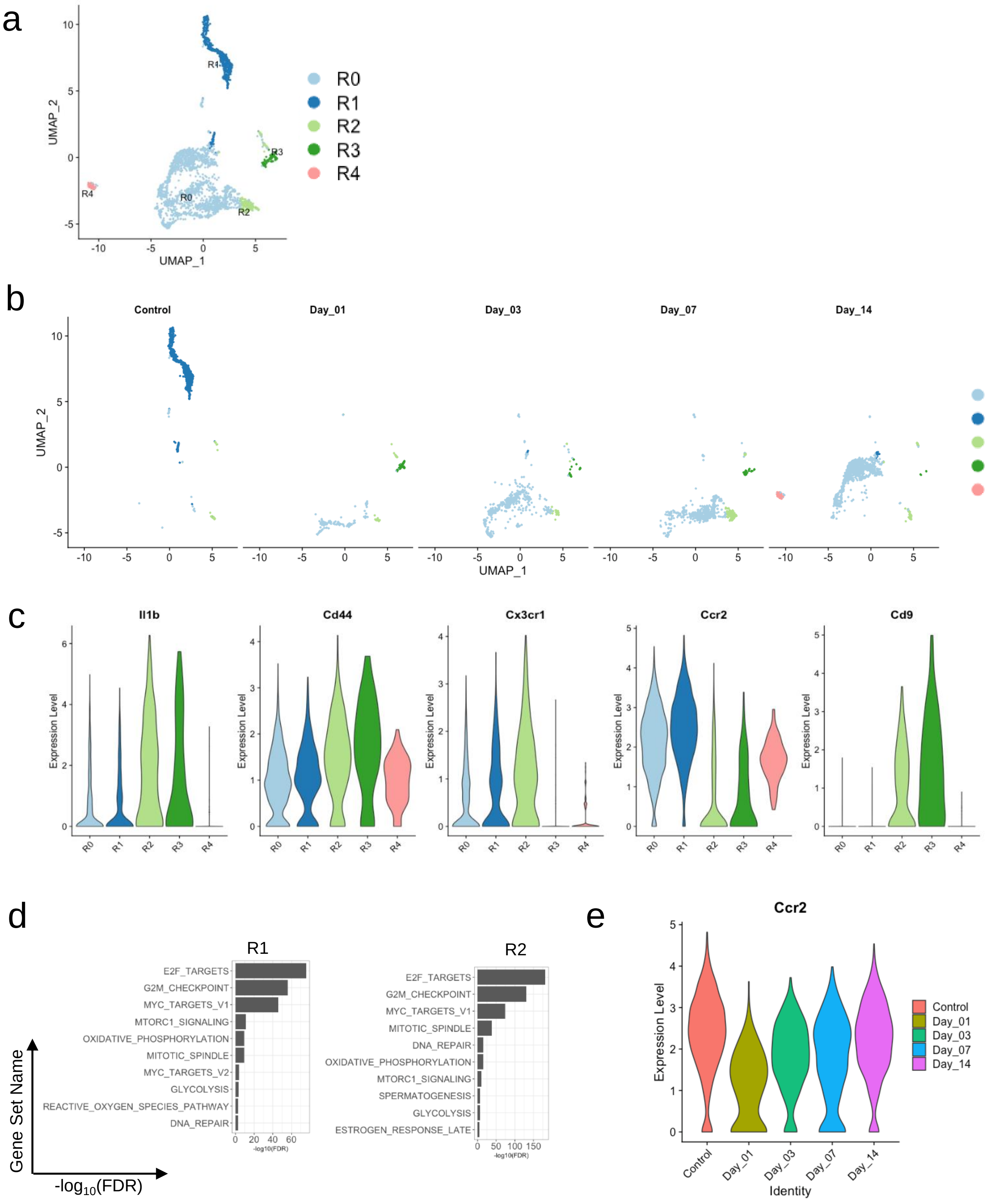

Supplementary Fig. 4. Cell-cycle and inflammatory signatures of cluster m1 and m3 *Cx3cr1+* cells. a) UMAP of re-analyzed m1 and m3 cells. b) UMAP showing clustering of m1 and m3 cells in naïve bone (control) and in interface tissue at each time point (days 1, 3, 7 and 14 post surgery). c) Violin plots of inflammation-related genes and macrophage subset markers in m1 and m3 cells. d) Pathway analysis using GSEA Hallmark gene set. e) Violin plot of Ccr2 expression and the indicated time points.

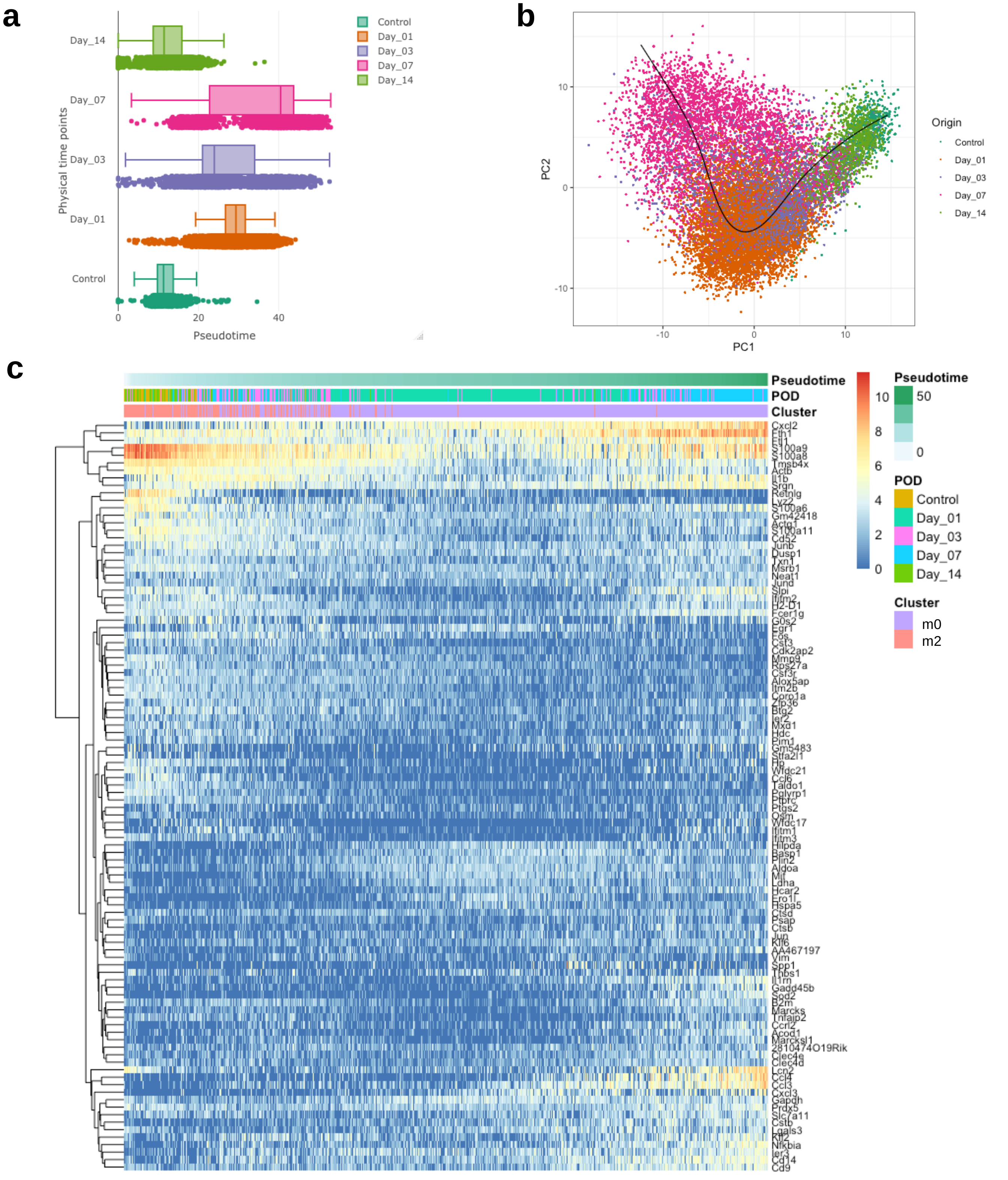

Fujii et al. Supplementary figure 5

Supplementary Figure 5. Pseudotime analysis of *Cd9*+ macrophages. a) Pseudtime values in each real time point. b) Principle curve of pseudotime values was projected on a PCA plot. Dots are grouped by physical time points. Black line shows pseudotime values starting from upper right end. c) Gene expression dynamics over pseudotime values was depicted in a heat map.

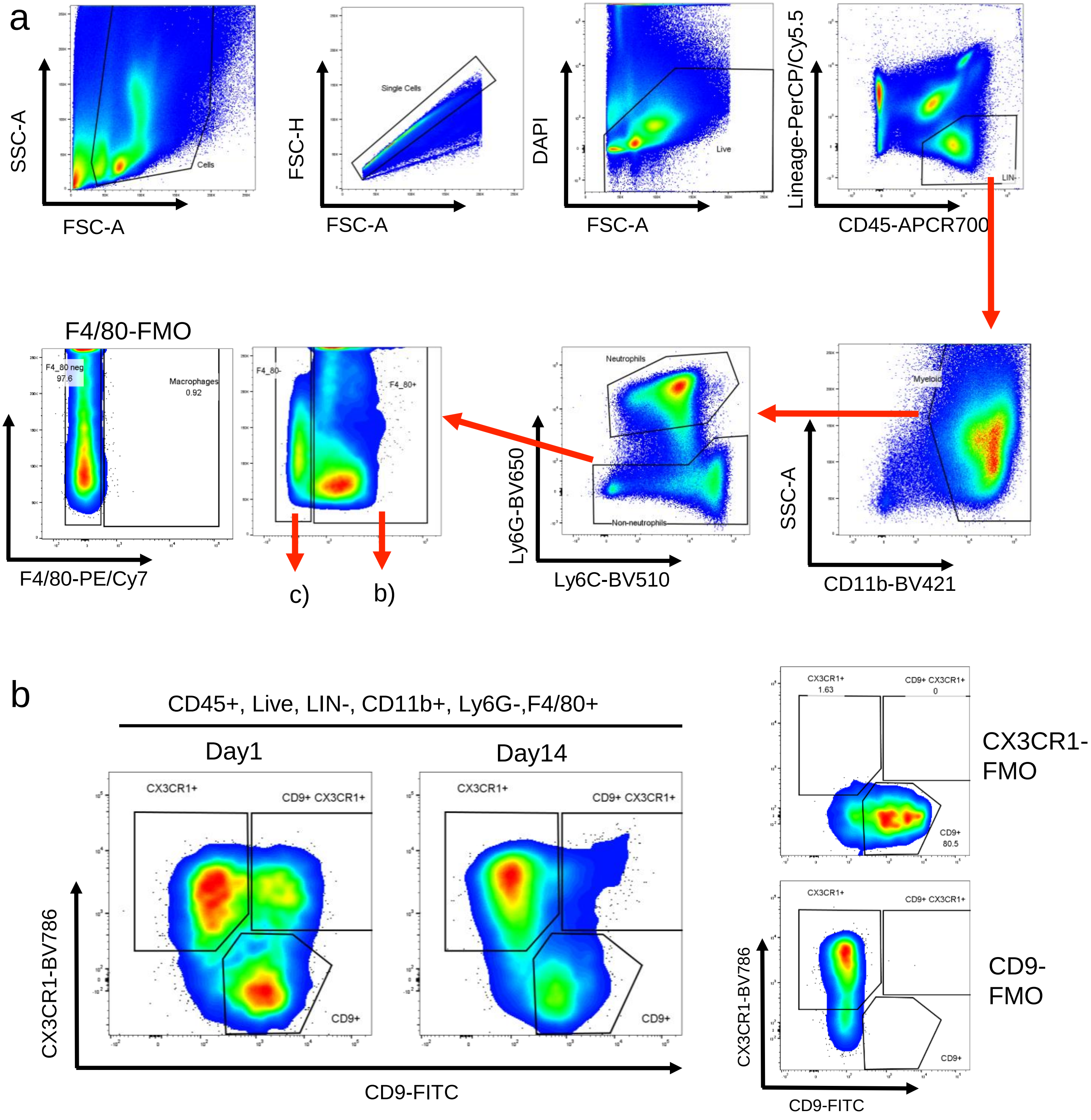

Fujii et al. Supplementary Figure 6

c

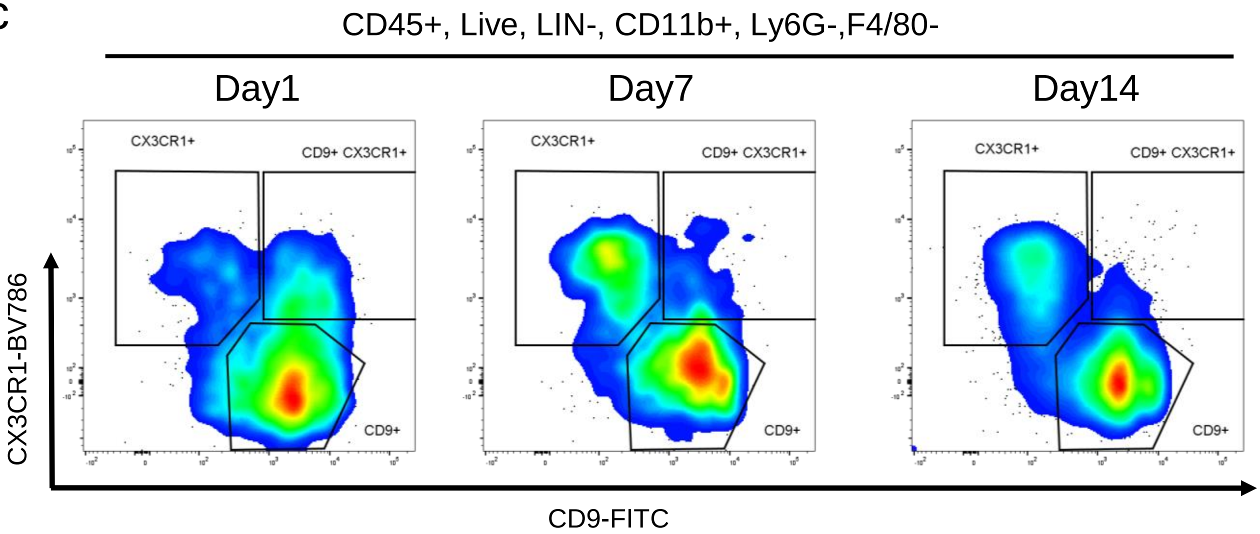

Supplementary Figure 6. Flow cytometric analysis of interface tissue.

a) Gating strategy. b) CD9 and CX3CR1 expressions in F4/80+ cells. c) CD9 and CX3CR1 expressions in F4/80- cells. Gatings of F4/80, CD9 and CX3CR1 were determined by Fluorescent minus one (FMO) staining. CD34, c-kit, Ter119, NK1.1, CD3e and B220 were used for lineage markers.

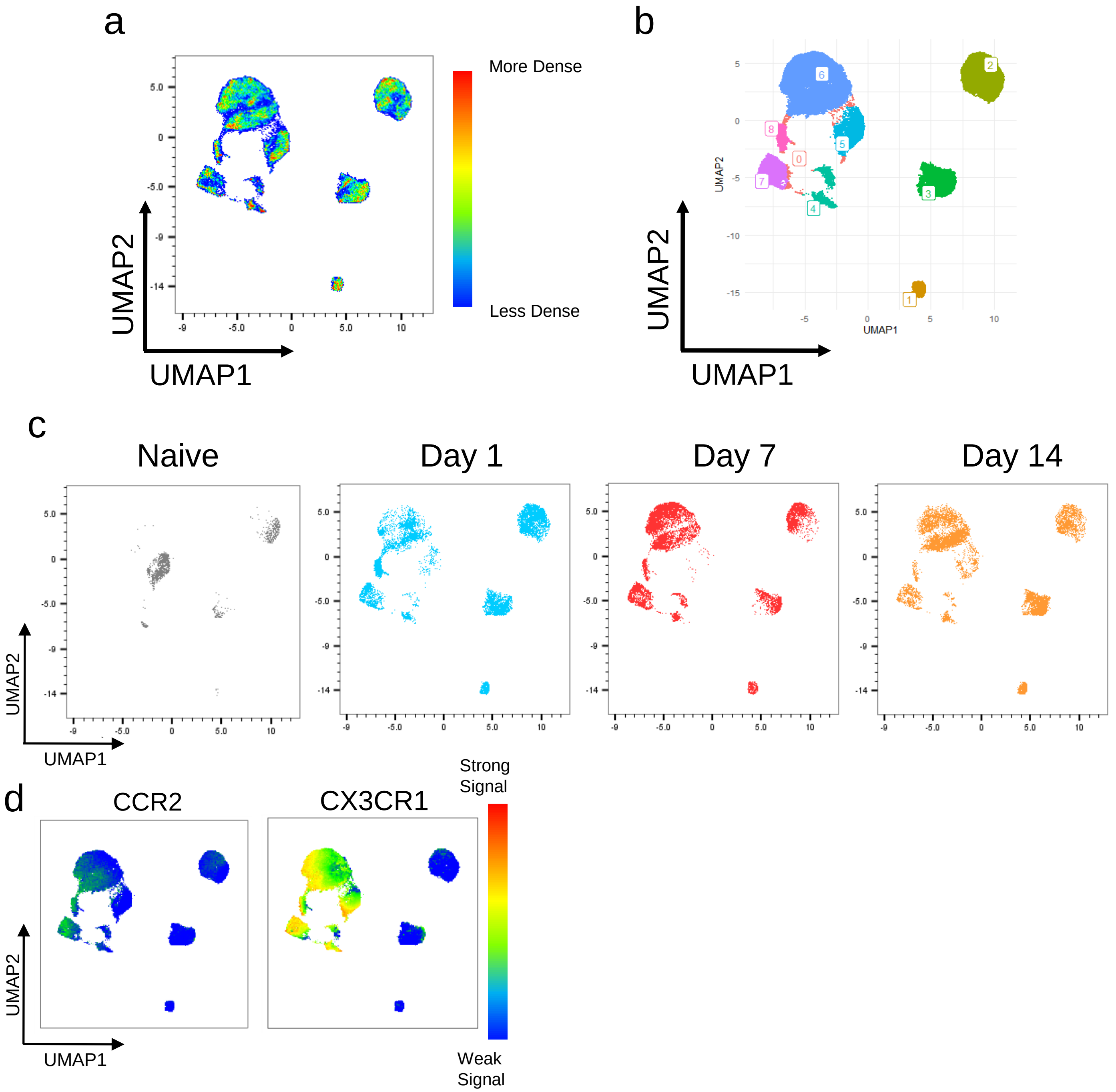

Supplementary Figure 7. Unbiased computational analysis of flow cytometry. Combined analysis of POD 1, 7, and 14 interface tissues contralateral bone, and naïve bone cells using antibodies against CD45, CD11b, CD11c, CD14 F4/80, CD64, CCR2, CX3CR1, CD9, CSF1R, Ly6C, Ly6G, MHC class II and lineage markers and monocyte/macrophage lineage cells (CD45+LIN-CD11b+Lyg6-F4/80+) were plotted on UMAP space. a) The expression levels of all markers in live CD45+lineage-CD11b+ cells from naïve bone marrow, contralateral bone marrow, and interface tissue of day1, 7, and 14 were computed, projected, and clustered on UMAP space with pseudo-color cell density. Although both interface tissue cells and contralateral bone marrow cells differentially increased relative to naive bone marrow cells, most of cell populations were overlapped in a UMAP. b) Clustering of macrophages on UMAP defines eight F4/80+ macrophage clusters. c) UMAP projection of monocyte/macrophage lineage cells from control naïve bone/ bone marrow and from the interface tissue at the indicated time points. d) UMAP projection showing expression of CD9 and CX3CR1 in cell clusters.

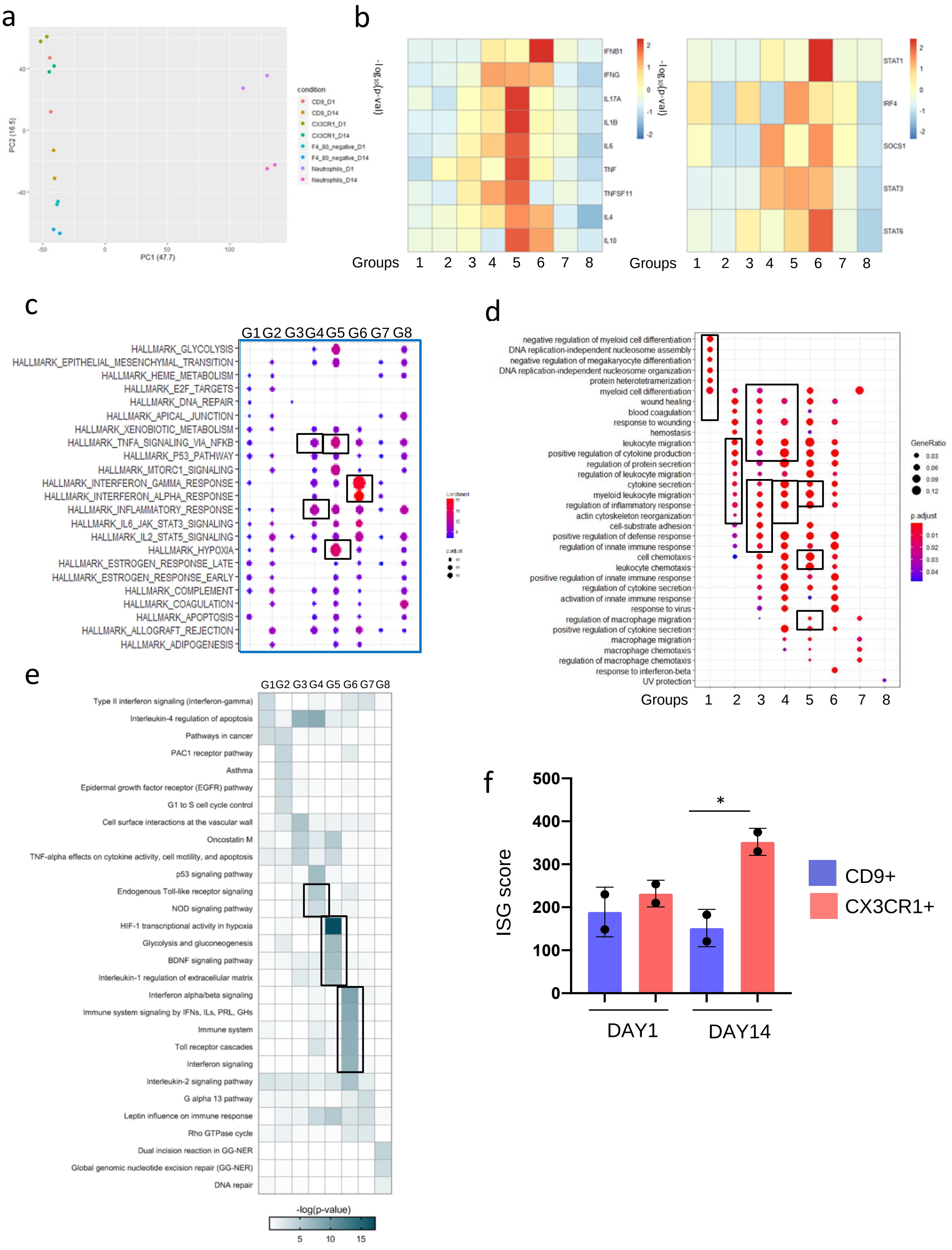

Fujii et al. Supplementary Figure 8

Supplementary Figure 8. Bulk RNA-seq analysis of myeloid cells in interface tissue. a) A PCA plot of CD11b+ myeloid cells b) Heat map showing cytokines (left panel) and interferon-stimulated genes (right panel) in the clusters defined in 5C. c) GSEA Hallmark gene set pathway analysis. d) Gene ontology analysis using clusterprofiler. e) ENRICHR pathway analysis f) Interferon-stimulated gene score (ISG score) of CD9+ macrophages and CX3CR1+ macrophages at POD 1 and 14. n=2. *: $p<0.05$, Welch’s t-test.

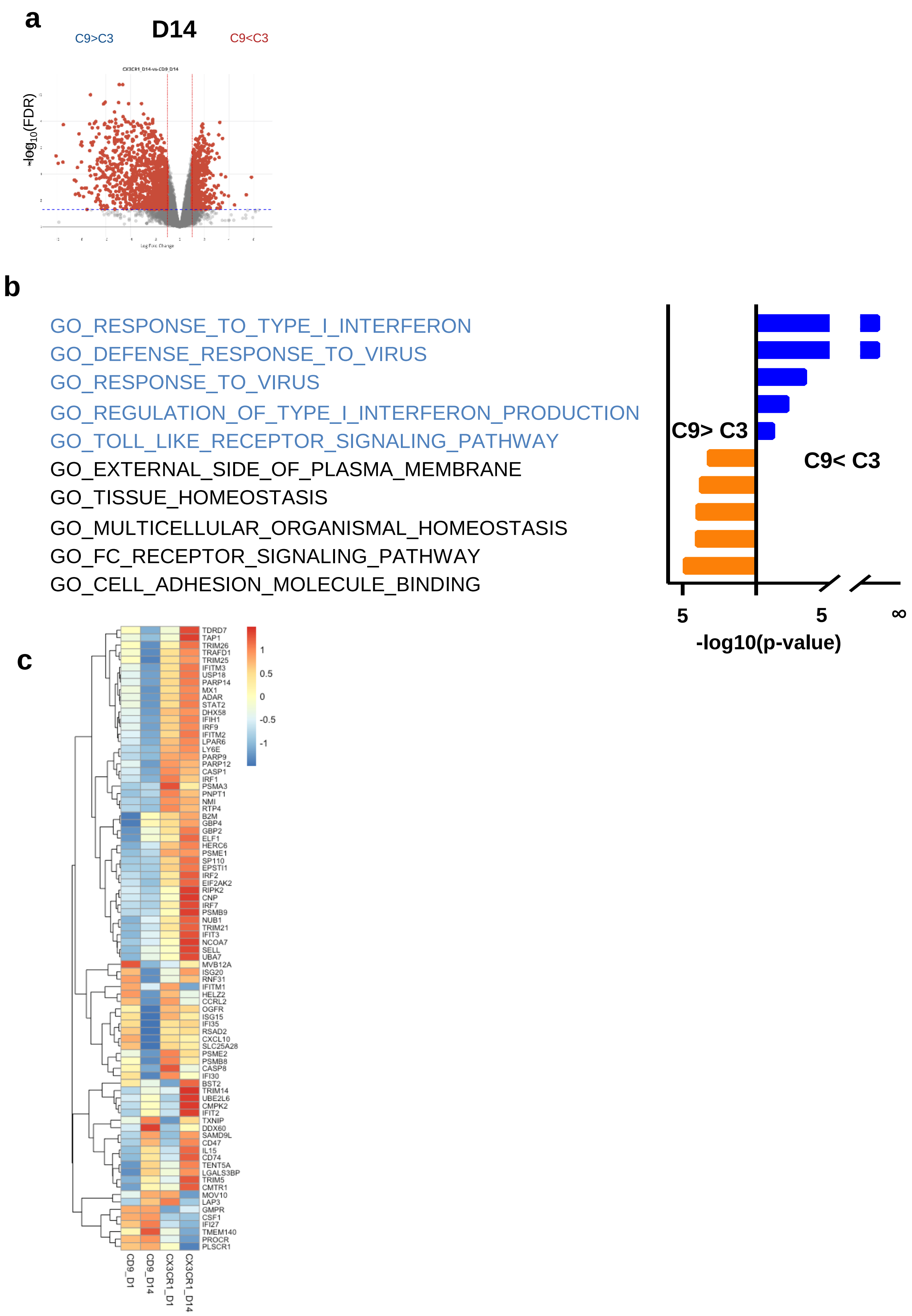

Supplementary Figure 9. Comparison of each population in interface tissue.
a) Volcano plot of RNA sequencing analysis of differentially expressed genes between CX3XR1+ macrophages (C3) and CD9+ macrophages (C9) on POD 14. b) GSEA GO pathway analysis. c) Heat map of individual ISG genes from GSEA Hallmark gene set. Averaged CPMs were z-score normalized.
