# Supplementary material for: Distinct Inflammatory Macrophage Populations Sequentially Infiltrate Bone‐to‐Tendon Interface Tissue After Anterior Cruciate Ligament (ACL) Reconstruction Surgery in Mice
Source: JBMR Plus. 2022 May 31;6(7):e10635. doi: 10.1002/jbm4.10635 (PMC9289991; doi:10.1002/jbm4.10635)
Supplement: Supplementary file 3 — Tables S1–S2. Supporting information [file JBM4-6-e10635-s001.docx]

Table S1.

The number of cells used for single-cell RNA sequencing.

|  | Total cell # | Mean reads/cell | Median genes/cell |
| --- | --- | --- | --- |
| Day 1 | 15339 | 22154 | 1189 |
| Day 3 | 9574 | 33826 | 1598 |
| Day 7 | 11724 | 27871 | 1378 |
| Day 14 | 10117 | 29436 | 1752 |

**Table S2.**

A list of antibodies used in flow cytometry.

| **Fluorochrome** | **Antigen** | **Cat#** | **Clone** | **Company** |
| --- | --- | --- | --- | --- |
| FITC | CD9 | 124808 | MZ3 | Biolegend |
| PE | CD14 | 123310 | Sa14-2 | Biolegend |
| PE/Dazzle594 | CSF1R | 135527 | ASF98 | Biolegend |
| PE/Cy7 | F4/80 | 123114 | BM8 | Biolegend |
| APC | CCR2 | 150628 | SA203G11 | Biolegend |
| APC R700 | CD45 | 565478 | 30-F11 | BD |
| BV421 | CD11b | 101235 | M1/70 | Biolegend |
| BV510 | Ly6C | 128033 | HK1.4 | Biolegend |
| BV605 | CD64 | 139323 | X54-5/7.1 | Biolegend |
| BV650 | Ly6G | 127641 | 1A8 | Biolegend |
| BV786 | CX3CR1 | 149029 | SA011F11 | Biolegend |
| BUV395 | MHC classII | 743876 | 2G9 | BD |
| BUV496 | DAPI | D1306 |  | Thermo Scientific |
| BUV737 | CD11c | 749039 | N418 | BD |
| Biotin | CD34 | 128604 | HM34 | Biolegend |
| Biotin | c-kit | 105804 | 2B8 | Biolegend |
| Biotin | Ter119 | 116204 | TER-119 | Biolegend |
| Biotin | NK1.1 | 108704 | PK136 | Biolegend |
| Biotin | CD3e | 100304 | 145-2C11 | Biolegend |
| Biotin | B220 | 103204 | RA3-6B2 | Biolegend |
| Streptavidin-PerCP/Cy5.5 |  | 405214 |  | Biolegend |
